# Supplementary material for: Intein-mediated temperature control for complete biosynthesis of sanguinarine and its halogenated derivatives in yeast
Source: Nat Commun. 2024 Jun 19;15:5238. doi: 10.1038/s41467-024-49554-w (PMC11186835; doi:10.1038/s41467-024-49554-w)
Supplement: Supplementary file 1 — Supplementary Information [file 41467_2024_49554_MOESM1_ESM.docx]

**S****upporting Information**

**Intein-mediated temperature control for complete biosynthesis of sanguinarine and its halogenated derivatives in yeast**

Yuanwei Gou^1,2^, Dongfang Li^2^, Minghui Zhao^1,2^, Mengxin Li^1^, Jiaojiao Zhang^2^, Yilian Zhou^2^, Feng Xiao^2^, Gaofei Liu^2^, Haote Ding^1,2^, Chenfan Sun^2^, Cuifang Ye^1^, Chang Dong^2^, Jucan Gao^2^, Di Gao^1^, Zehua Bao^1,2^, Lei Huang^1,2^, Zhinan Xu^1^, Jiazhang Lian^1,2^*

^1^ Key Laboratory of Biomass Chemical Engineering of Ministry of Education, College of Chemical and Biological Engineering, Zhejiang University, Hangzhou, 310027, China

^2^ Hangzhou Global Scientific and Technological Innovation Center, Zhejiang University, Hangzhou, 310027, China

***Correspondence**

Prof. Jiazhang Lian

Key Laboratory of Biomass Chemical Engineering of Ministry of Education, College of Chemical and Biological Engineering, Zhejiang University, Hangzhou, 310027, China

Email: [jzlian@zju.edu.cn](mailto:jzlian@zju.edu.cn)


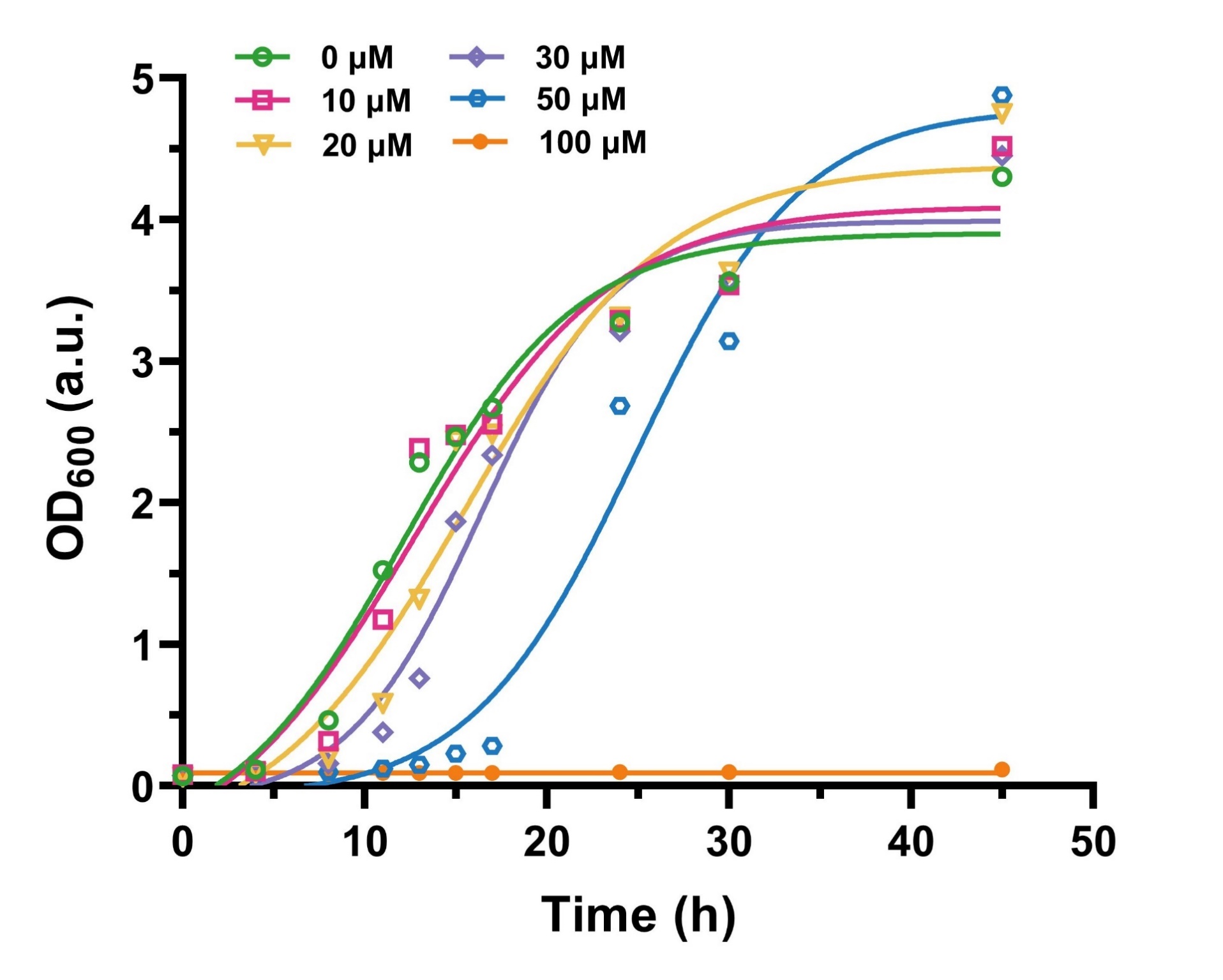
**Supplementary Figures**

**Supplementary Fig. 1** Growth curves of BY4741 in the presence of 0-100 µM sanguinarine. Higher concentrations of sanguinarine resulted in significant growth inhibition. Data are presented as mean ± s.d. (n = 3 biologically independent samples). Source data are provided as a Source Data file.


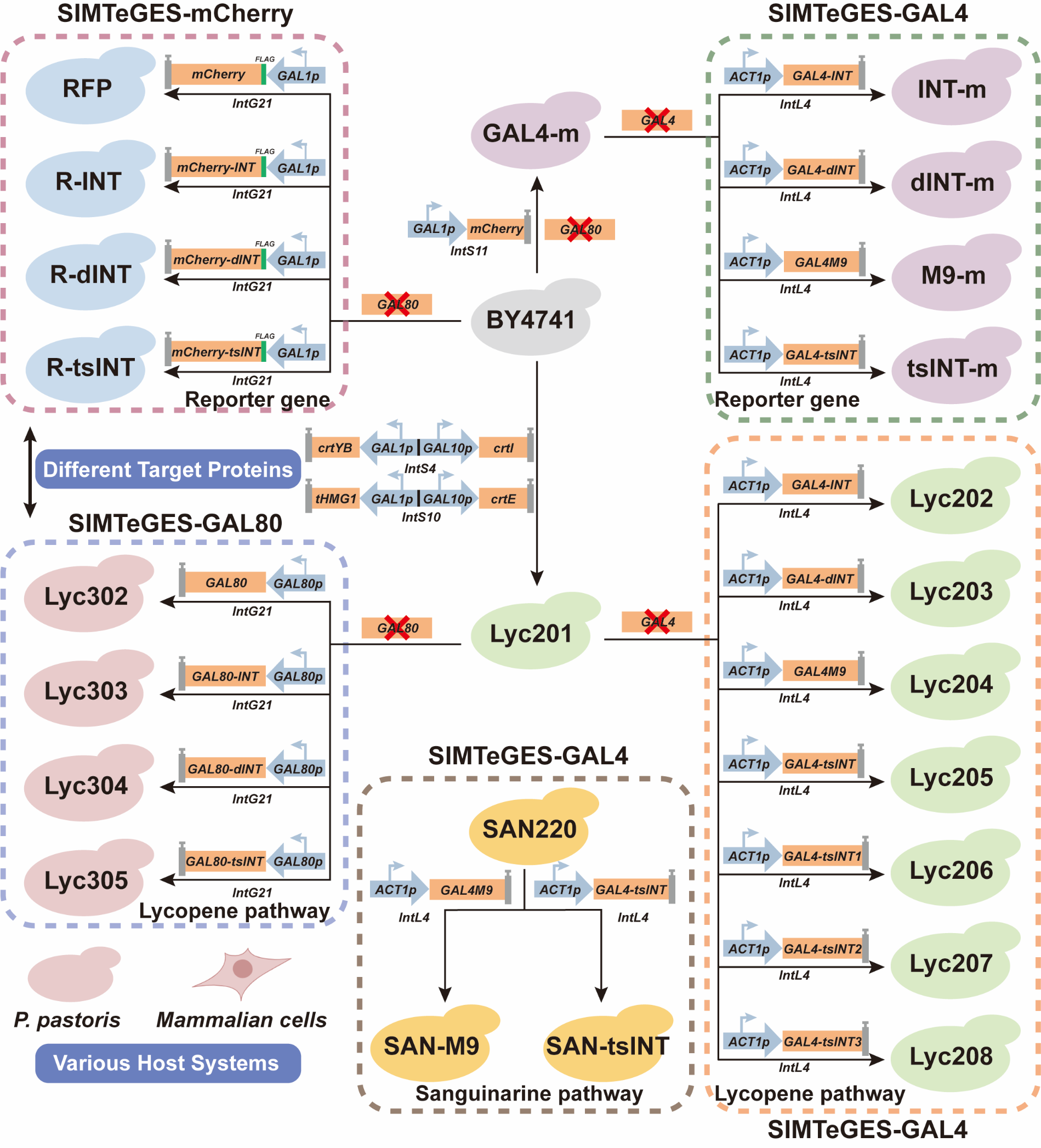
**Supplementary Fig. 2** Construction procedures for SIMTeGES-related strains. Detailed depiction of the integrated gene cassette with the utilized promoters and their corresponding integration sites. Detailed information regarding the integration sites can be found in Supplementary Table 4.


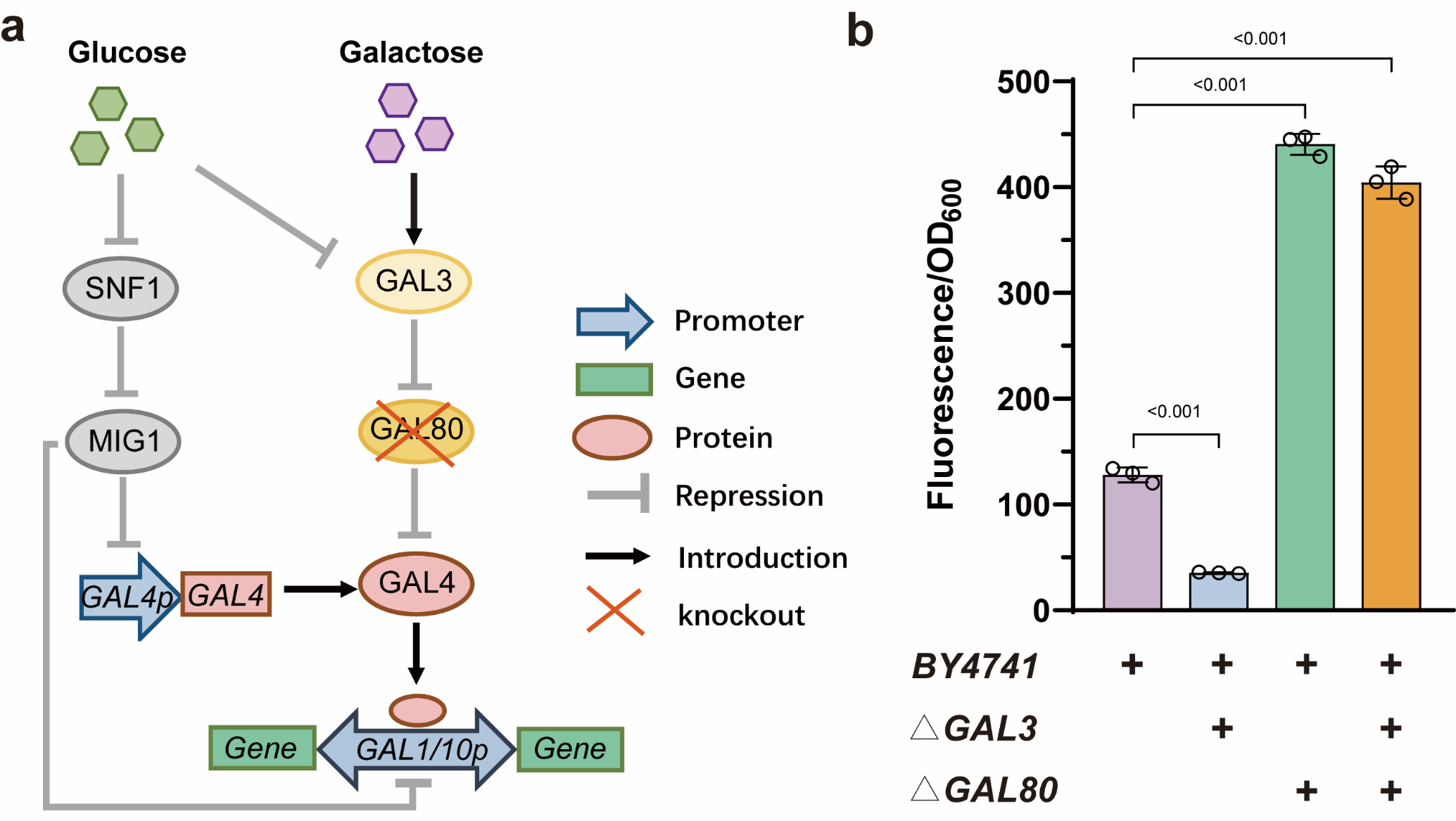
**Supplementary Fig. 3** Overview of the GAL regulatory system in *S. cerevisiae.* (a) Transcription initiated by the *GAL* promoters (*GAL1p*, *GAL10p*, *GAL2p*, and *GAL7p*) is inhibited in the presence of glucose and expression begins only when glucose is depleted. (b) When galactose served as the carbon source, the deletion of the galactose signal transducer GAL3 prevented the initiation of genes regulated by the *GAL* promoters. Conversely, the absence of GAL80 results in sustained high-level expression of the GAL system, with the activity of the transcriptional activator GAL4 directly determining the functionality of the *GAL* promoters. Data are presented as mean ± s.d. (n = 3 biologically independent samples). Significance was calculated using one-way ANOVA followed by Tukey’s multiple comparisons test. Source data are provided as a Source Data file.


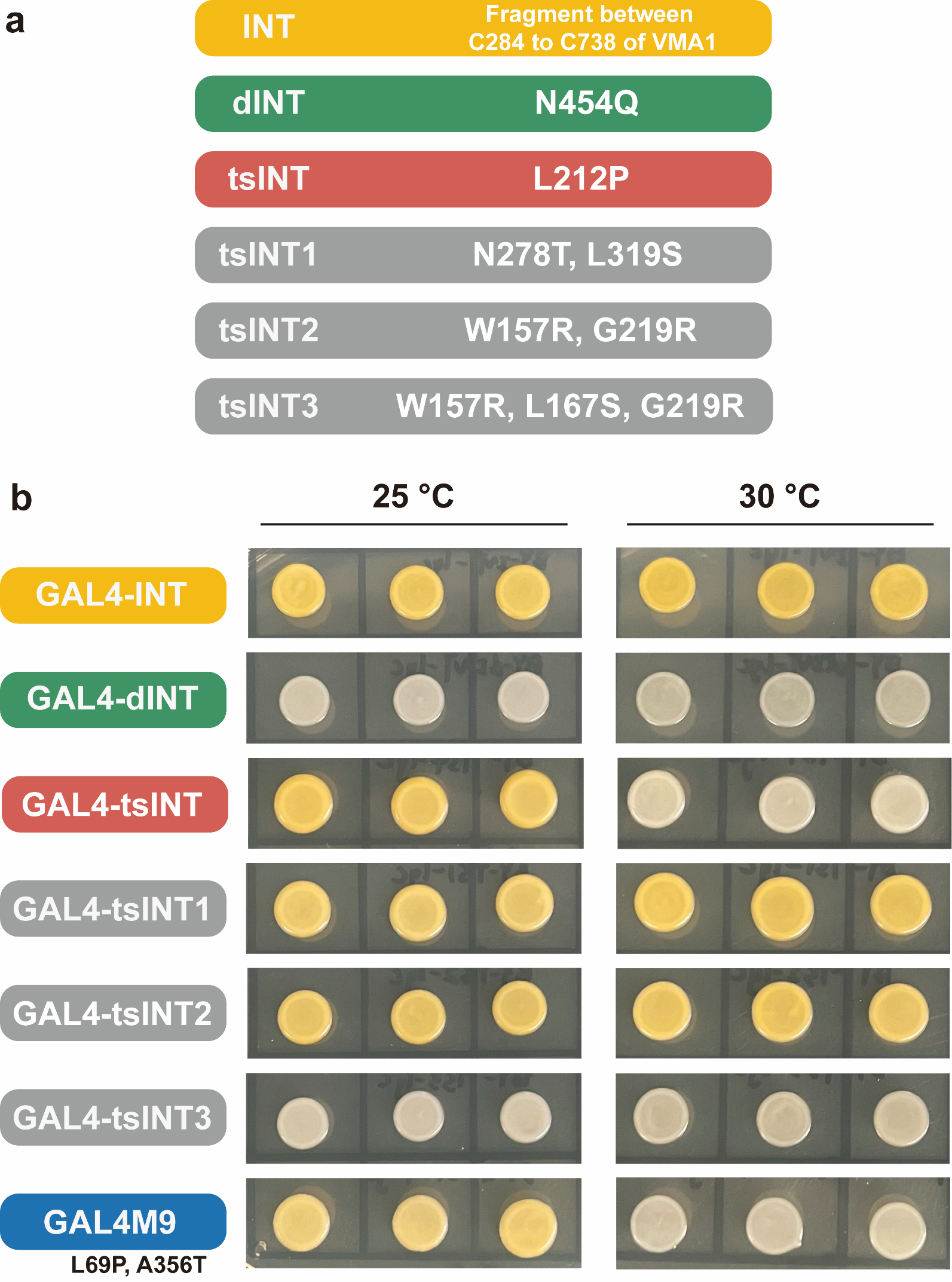
**Supplementary Fig. 4** Characterization of temperature-sensitive intein variants. (a) Sequence features of intein and intein variants. (b) The colored metabolite lycopene serves as a reporter system, reflecting GAL4 activity for screening appropriate intein variants. Three single clones for each strain were simultaneously spotted onto two YPG plates and incubated at 25 °C and 30 °C for 2 days, respectively. Source data are provided as a Source Data file.
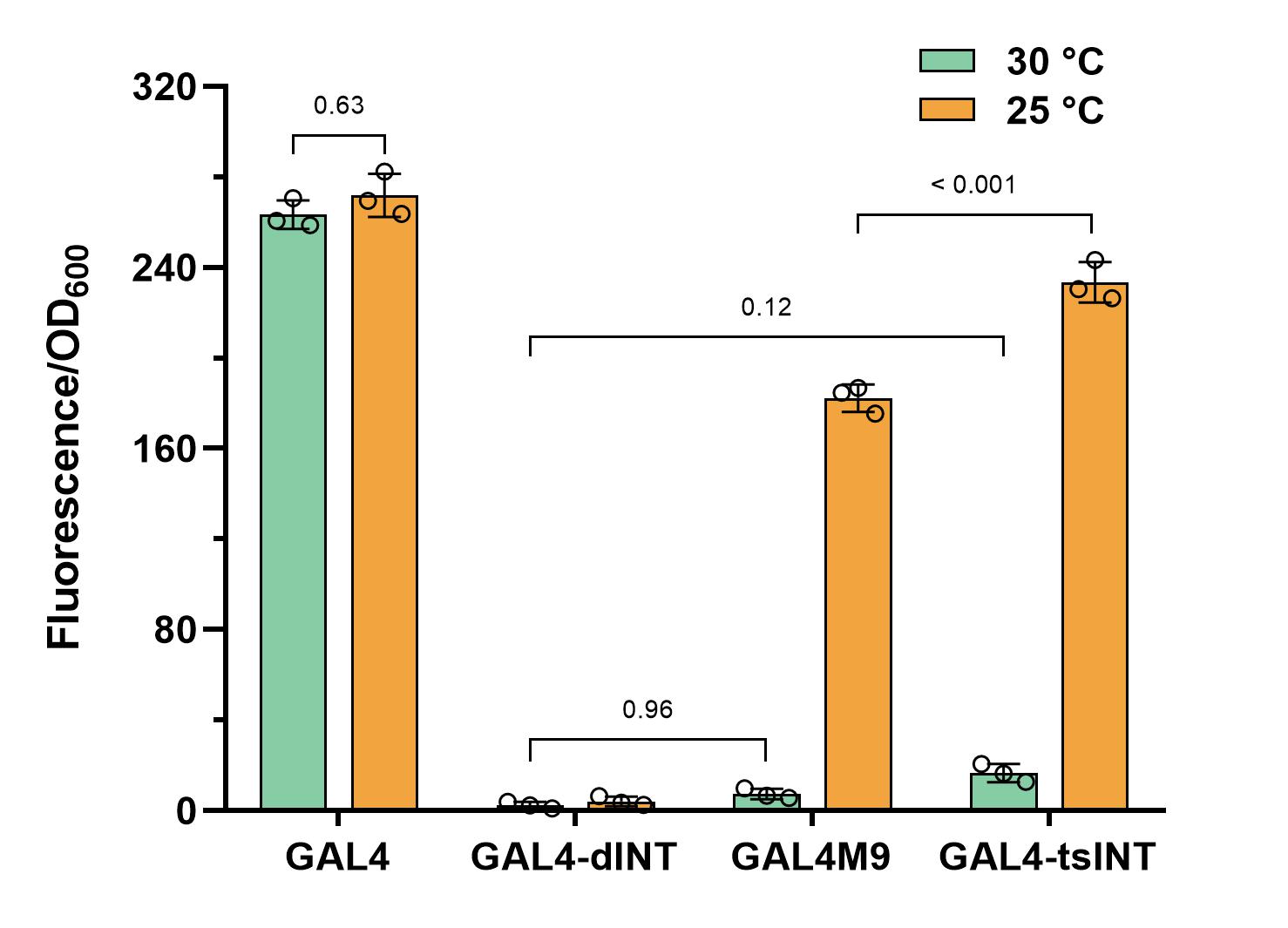
**Supplementary Fig. 5** Evaluation of SIMTeGES-GAL4 for temperature-controlled expression of *GAL1p-mCherry*. Strains were cultured in SEG medium for 36 h and evaluated for the activity and leaky expression of the temperature-regulated system using a microplate reader. Results aligned with Fig. 1C. Data are presented as mean ± s.d. (n = 3 biologically independent samples). Significance was calculated using two-way ANOVA followed by Tukey’s multiple comparisons test. Source data are provided as a Source Data file.


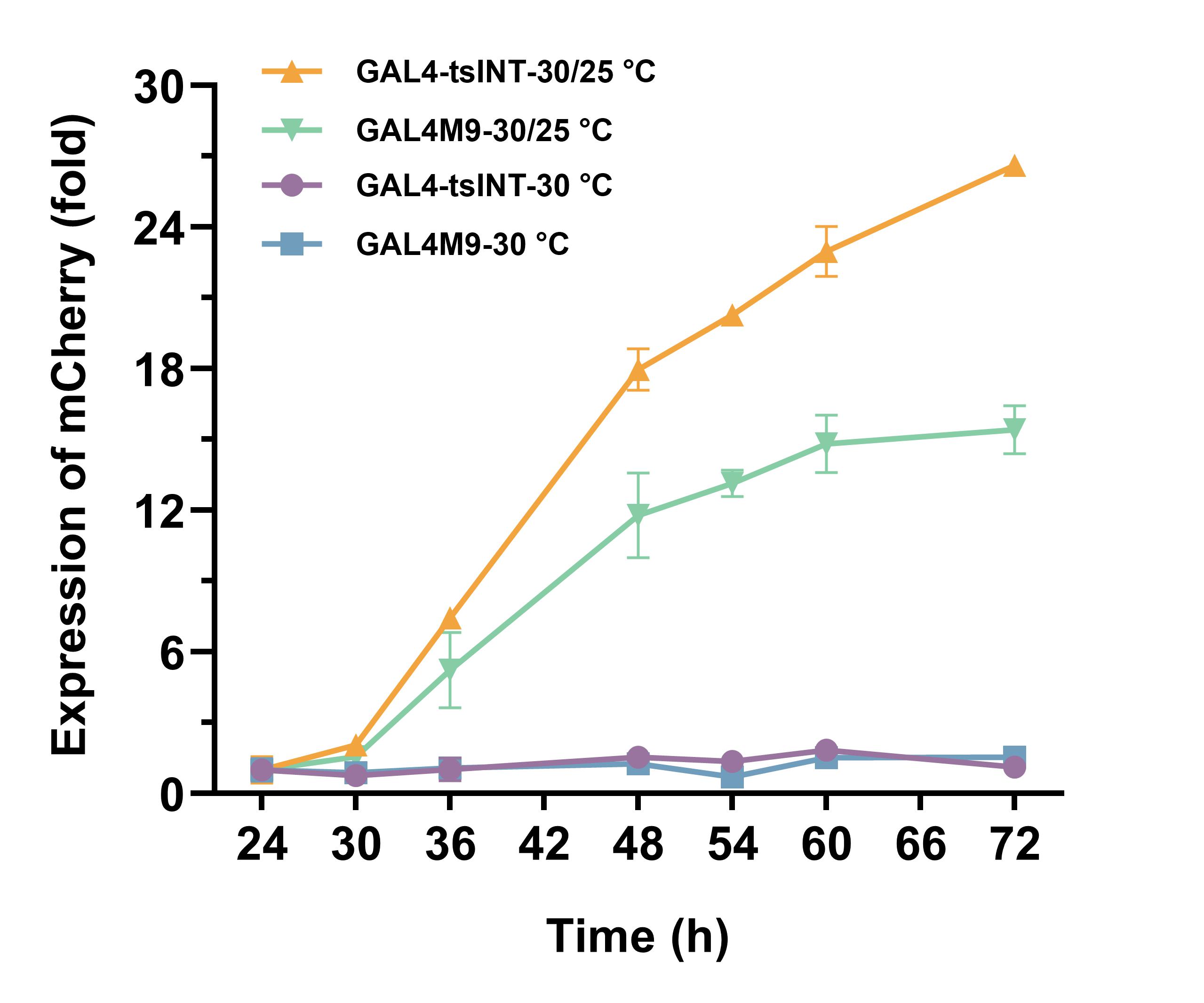
**Supplementary Fig. 6** Kinetics of SIMTeGES-GAL4, with the temperature-controlled expression of *GAL1p-mCherry* as the reporter gene. The fluorescence intensity of mCherry at 24 h (the time point for temperature shift) was set to 1. Data are presented as mean ± s.d. (n = 3 biologically independent samples). Source data are provided as a Source Data file.


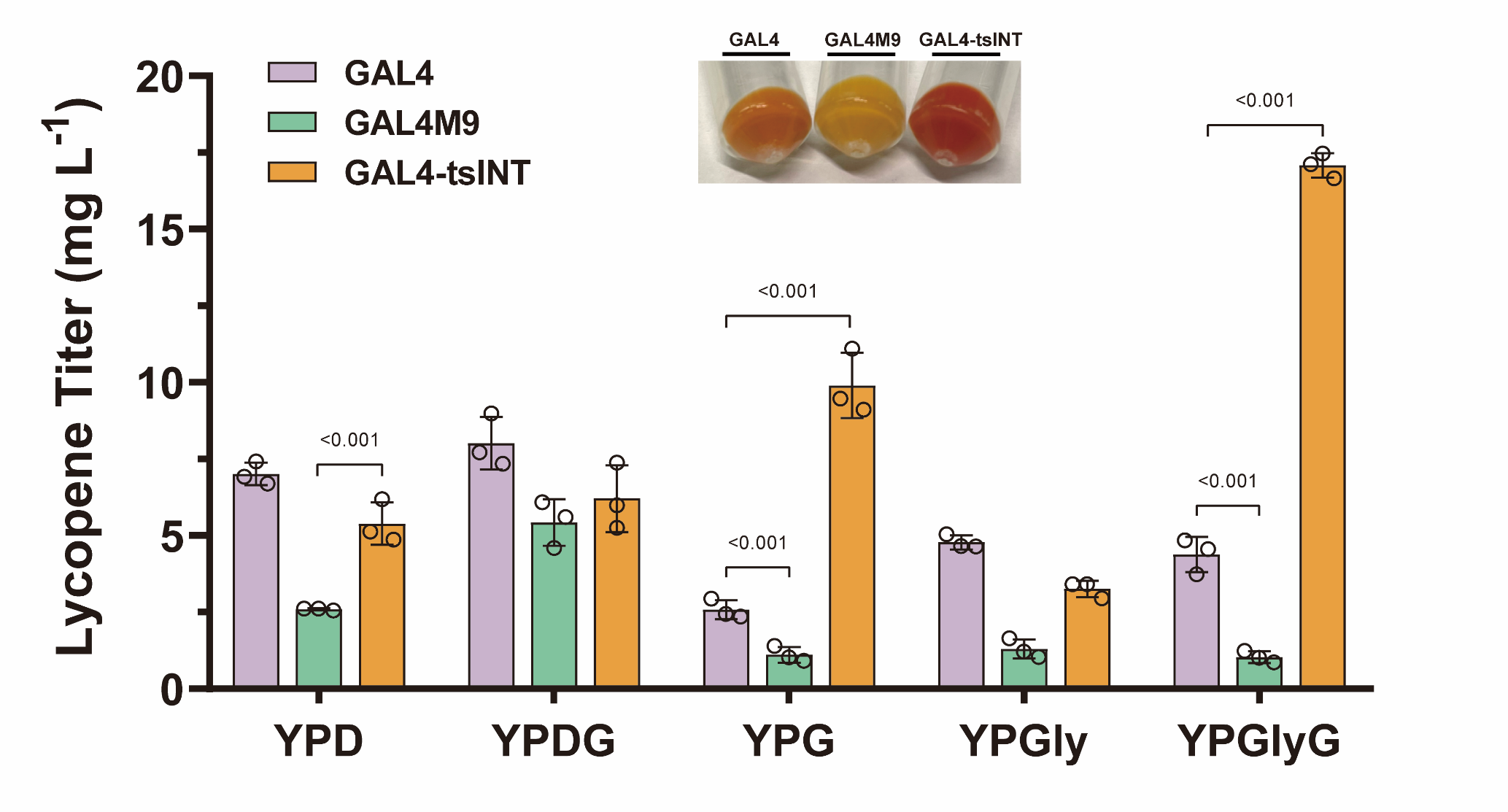
**Supplementary Fig. 7** Characterization of two temperature-responsive GAL regulation systems in the lycopene biosynthesis pathway under different carbon source conditions. After growing the strains at 30 °C for 24 h, temperature was shifted to 25 °C, and the cultures were fermented for an additional three days before harvesting the cells for lycopene quantification. YPD, YPDG, YPG, YPGly, and YPGlyG denote different fermentation conditions in YP medium, with the following carbon sources: 20 g L^-1^ glucose, a combination of 20 g L^-1^ glucose and 10 g L^-1^ galactose, 20 g L^-1^ galactose, 20 g L^-1^ glycerol, and a combination of 10 g L^-1^ glycerol and 20 g L^-1^ galactose, respectively. Data are presented as mean ± s.d. (n = 3 biologically independent samples). Significance was calculated using two-way ANOVA followed by Tukey’s multiple comparisons test. Source data are provided as a Source Data file.


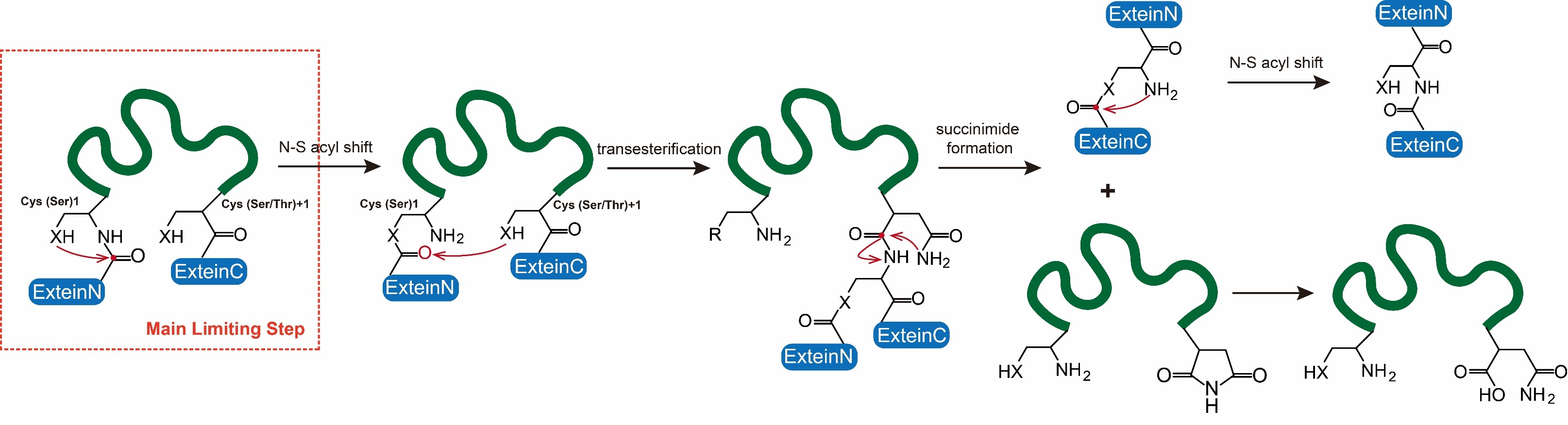
**Supplementary Fig. 8** Schematic illustrating the splicing mechanism of inteins, involving four principal steps: (1) N-O or N-S acyl rearrangement to form a linear ester intermediate, (2) reversible formation of a branched ester intermediate, (3) cleavage of the branched ester intermediate to yield an excised intein with a *C-*terminal aminosuccinimide residue and the two exteins joined by an ester bond, and (4) spontaneous hydrolysis of the aminosuccinimide residue and rearrangement of the ester linking the exteins to the more stable amide bond.


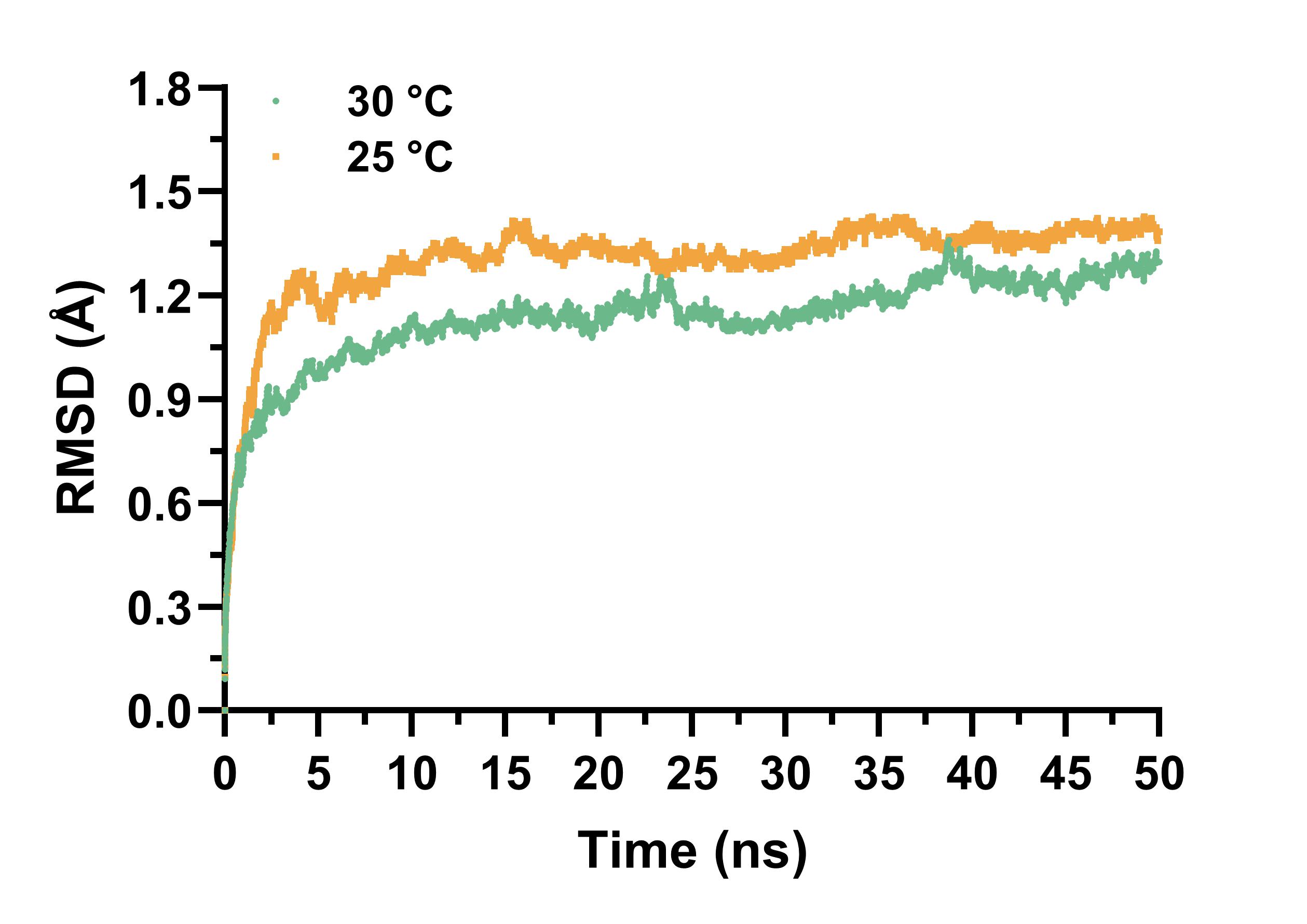
**Supplementary Fig. 9** Root-mean-square deviations (RMSD) values of GAL4-tsINT during MD simulation at 30 °C and 25 °C. Source data are provided as a Source Data file.


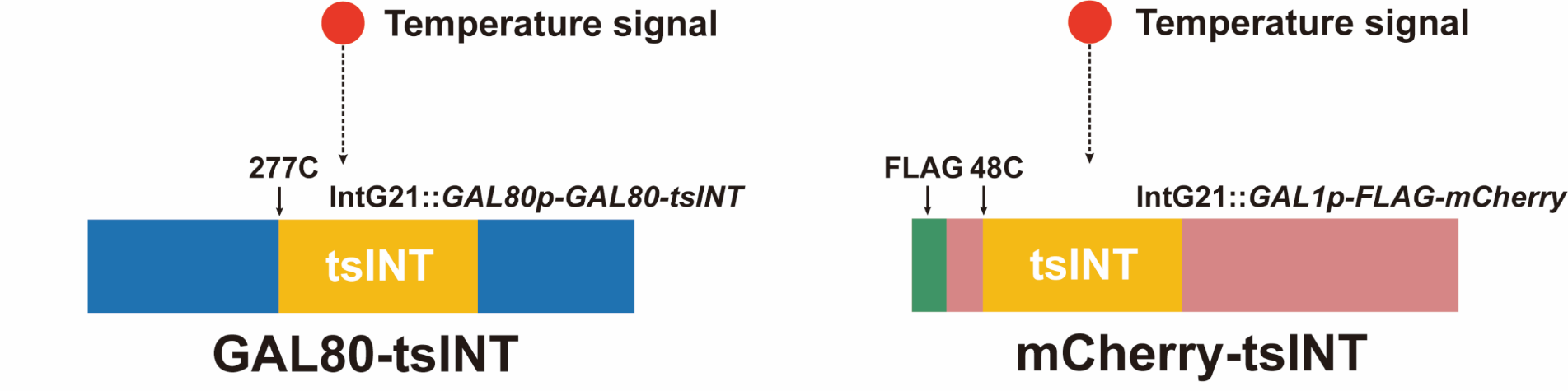


**Supplementary Fig. 10** Schematic diagrams for SIMTeGES-GAL80 and SIMTeGES-mCherry.


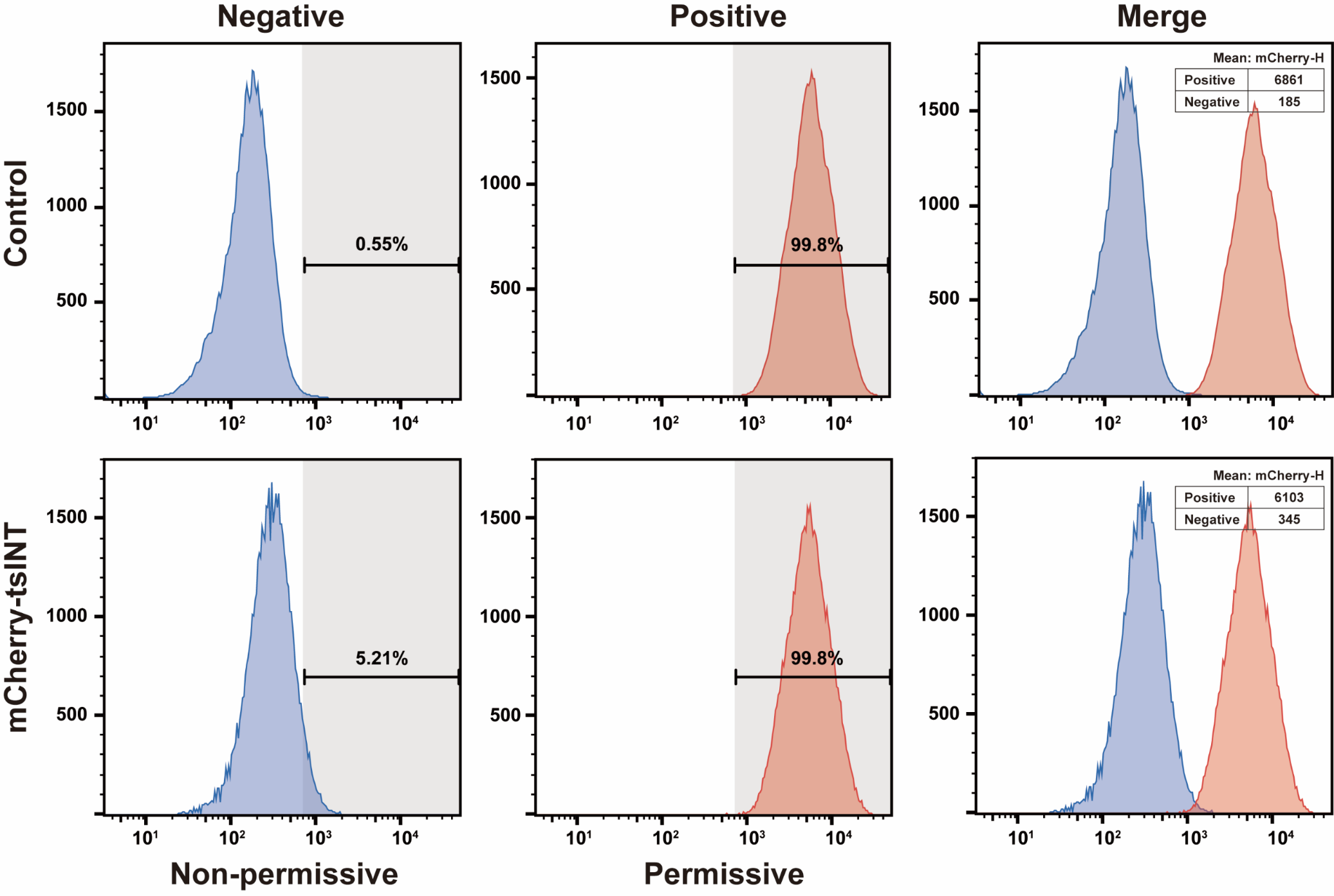
**Supplementary Fig. 11** Flow cytometry to further investigate the activity and leaky expression of mCherry-tsINT.


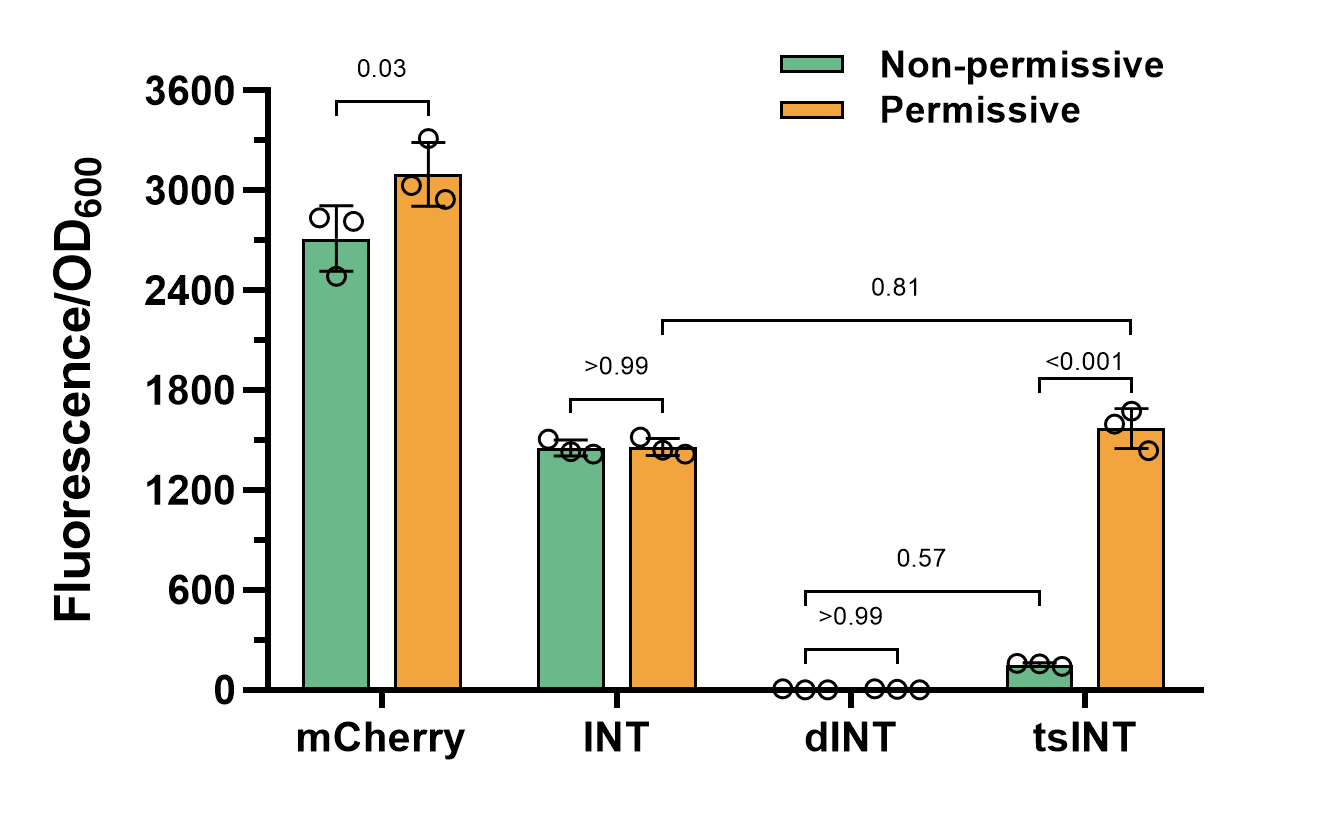
**Supplementary Fig. 12** Validation of temperature-dependent splicing inteins universality in *Pichia pastoris*. Strains cultured in SED medium for 24 h, followed by measurement of fluorescence intensity and cell densities (OD_600_) using microplate reader. Data are presented as mean ± s.d. (n = 3 biologically independent samples). Significance was calculated using two-way ANOVA followed by Tukey’s multiple comparisons test. Source data are provided as a Source Data file.


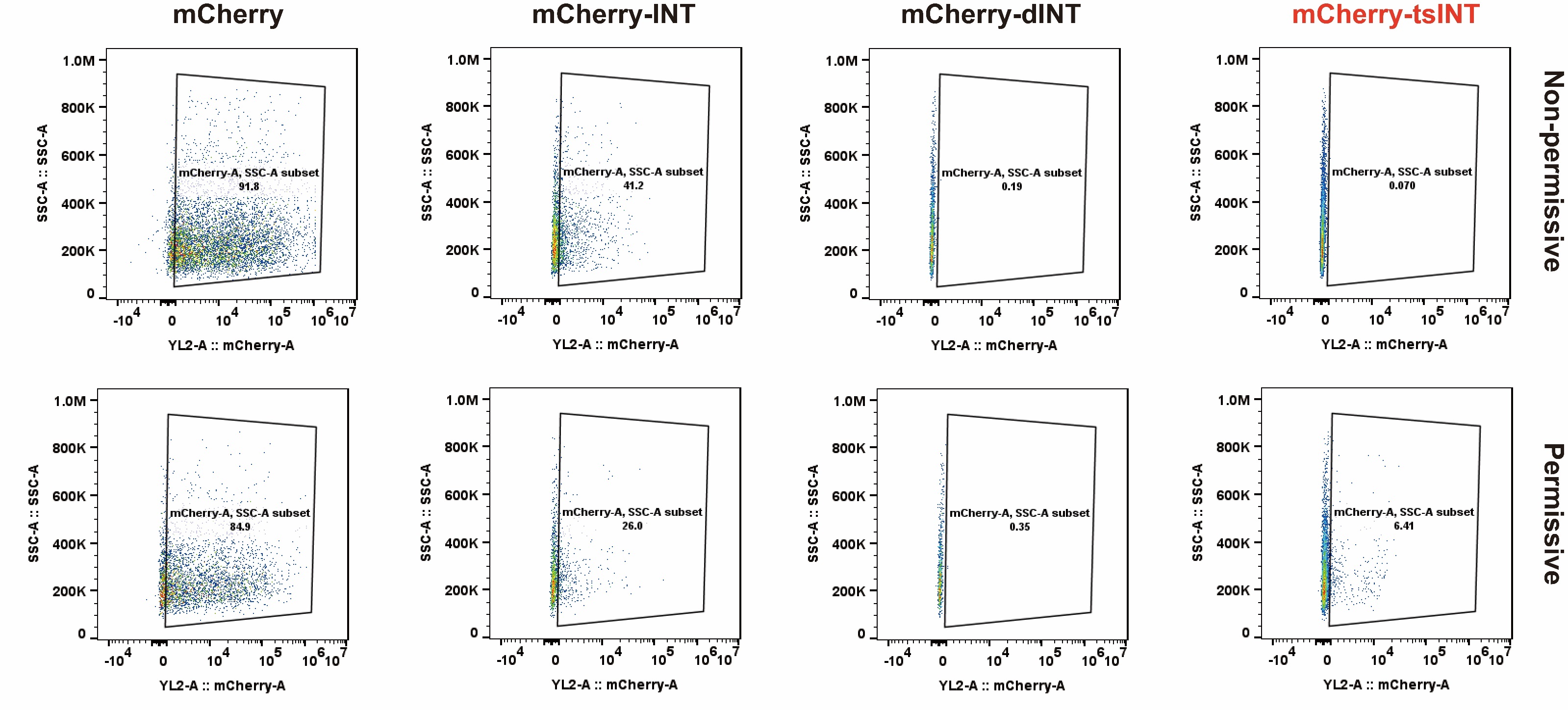
**Supplementary Fig. 13** Validation of temperature-dependent splicing inteins universality in mammalian cells. HEK293T cells were transfected and cultured under varied temperature conditions for 48 h and subsequently analyzed using flow cytometry.


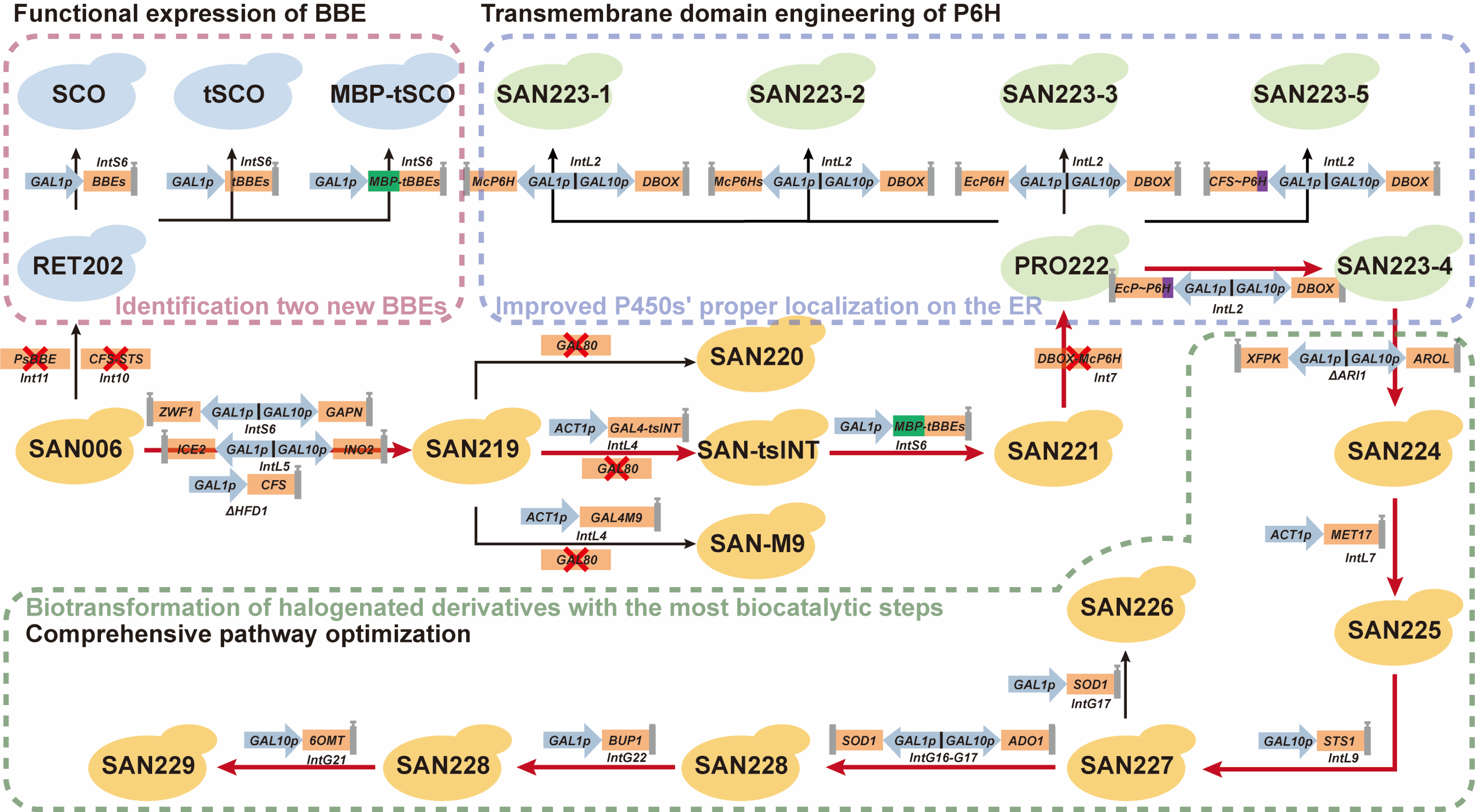
**Supplementary Fig. 14** Construction procedures for sanguinrine bioproduction-related strains. Detailed depiction of the integrated gene expression cassettes with the promoters and their corresponding integration sites. Detailed information regarding the integration sites can be found in Supplementary Table 4.


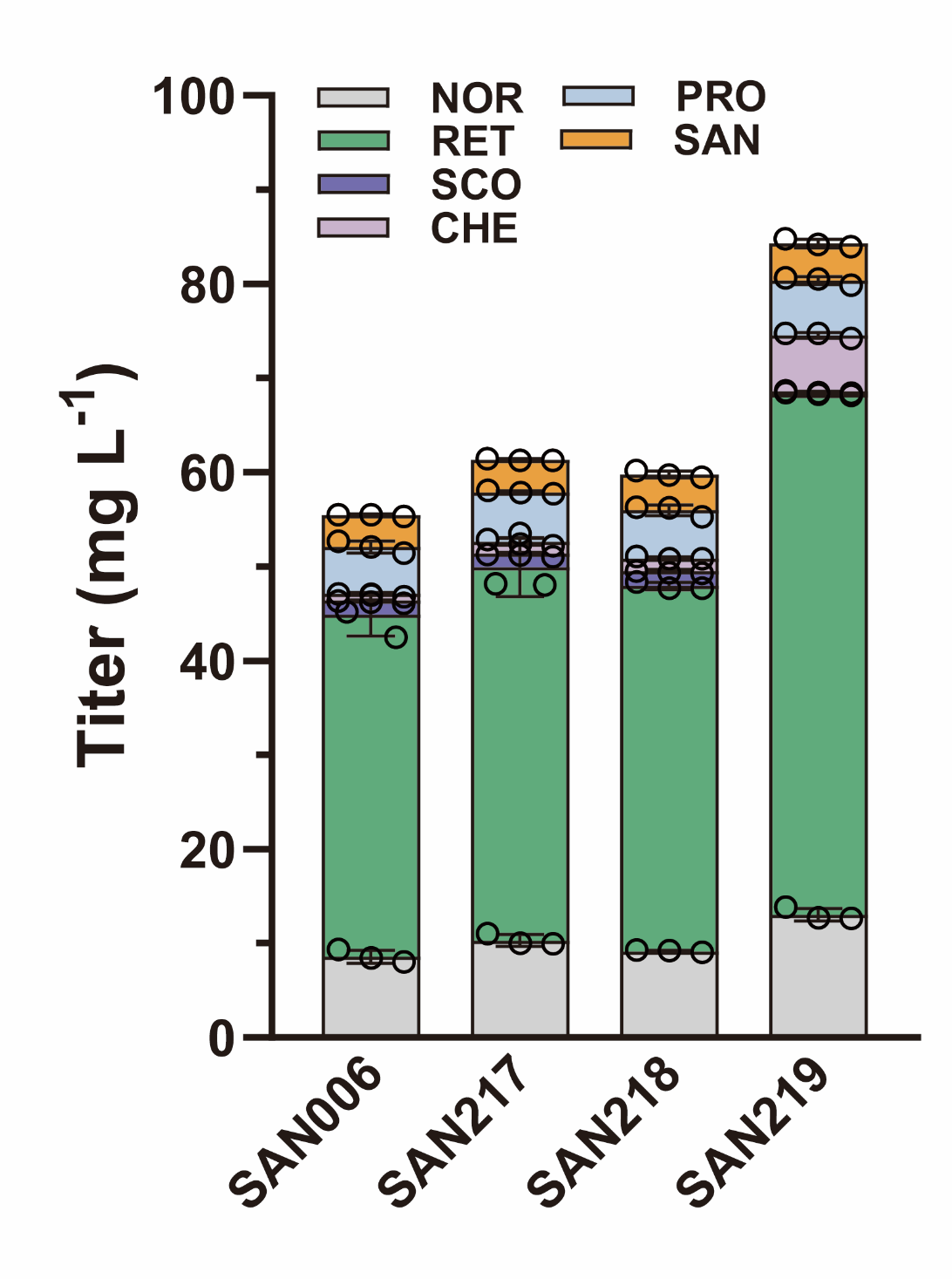
**Supplementary Fig. 15** Optimization of cellular microenvironment for functional expression and activity of cytochrome P450s. After 24 h of growth in SED medium, cells of SAN006-SAN219 strains were harvested and resuspended in SEG medium for a 36-h fermentation. The accumulation of intermediates and products in the fermentation broth was subsequently assessed using LC-QQQ. Data are presented as mean ± s.d. (n = 3 biologically independent samples). Source data are provided as a Source Data file.


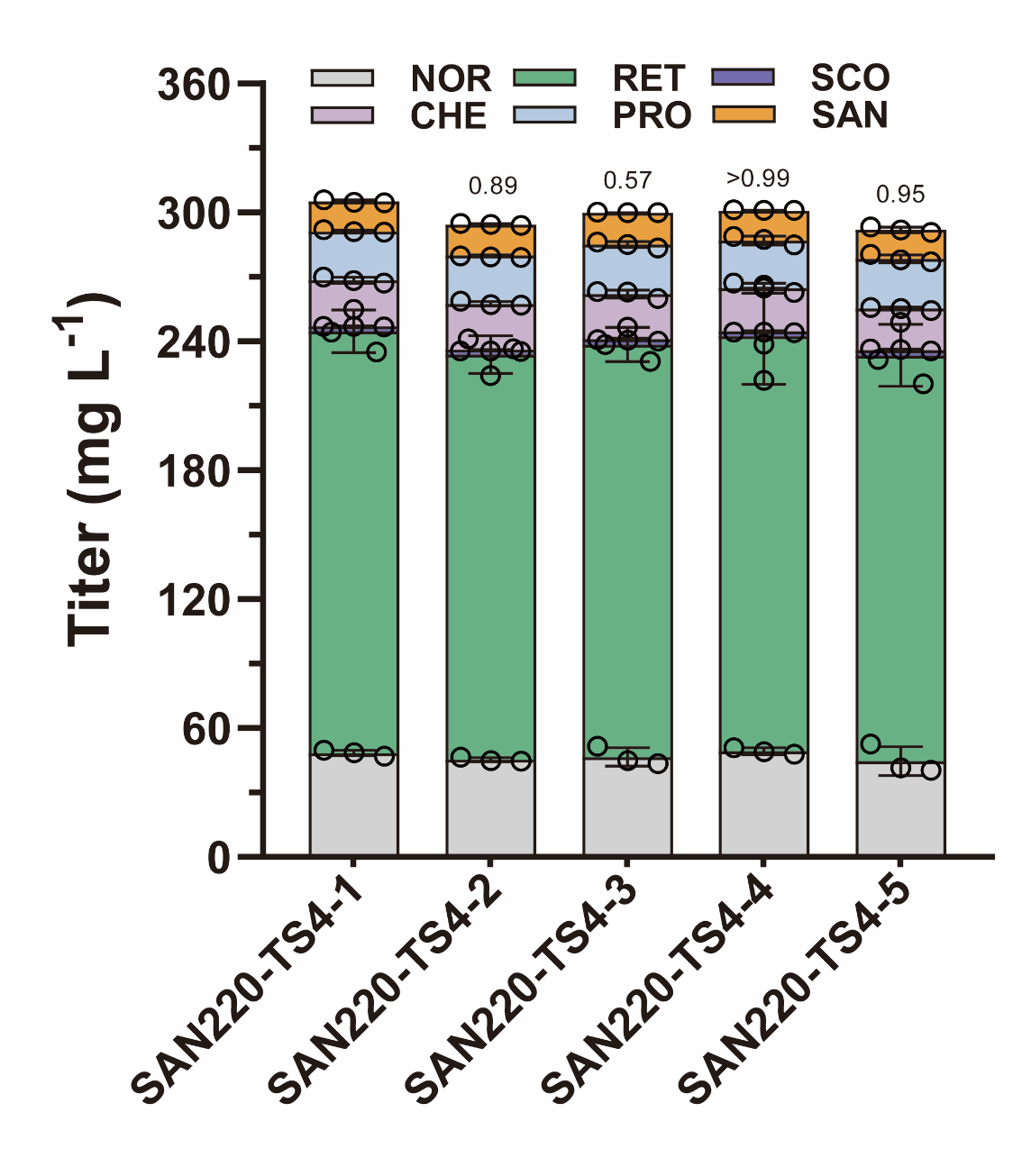
**Supplementary Fig. 16** Evaluation of the stability of sanguinarine-producing strain SAN220-tsINT. After five serial transfers in YPD medium, five independent colonies were randomly picked for sanguinarine fermentation and quantification. The strain stability was indicated by the variation in the production of sanguinarine and pathway intermediates. Data are presented as mean ± s.d. (n = 3 biologically independent samples). Significance was calculated using two-way ANOVA followed by Tukey’s multiple comparisons test. Source data are provided as a Source Data file.


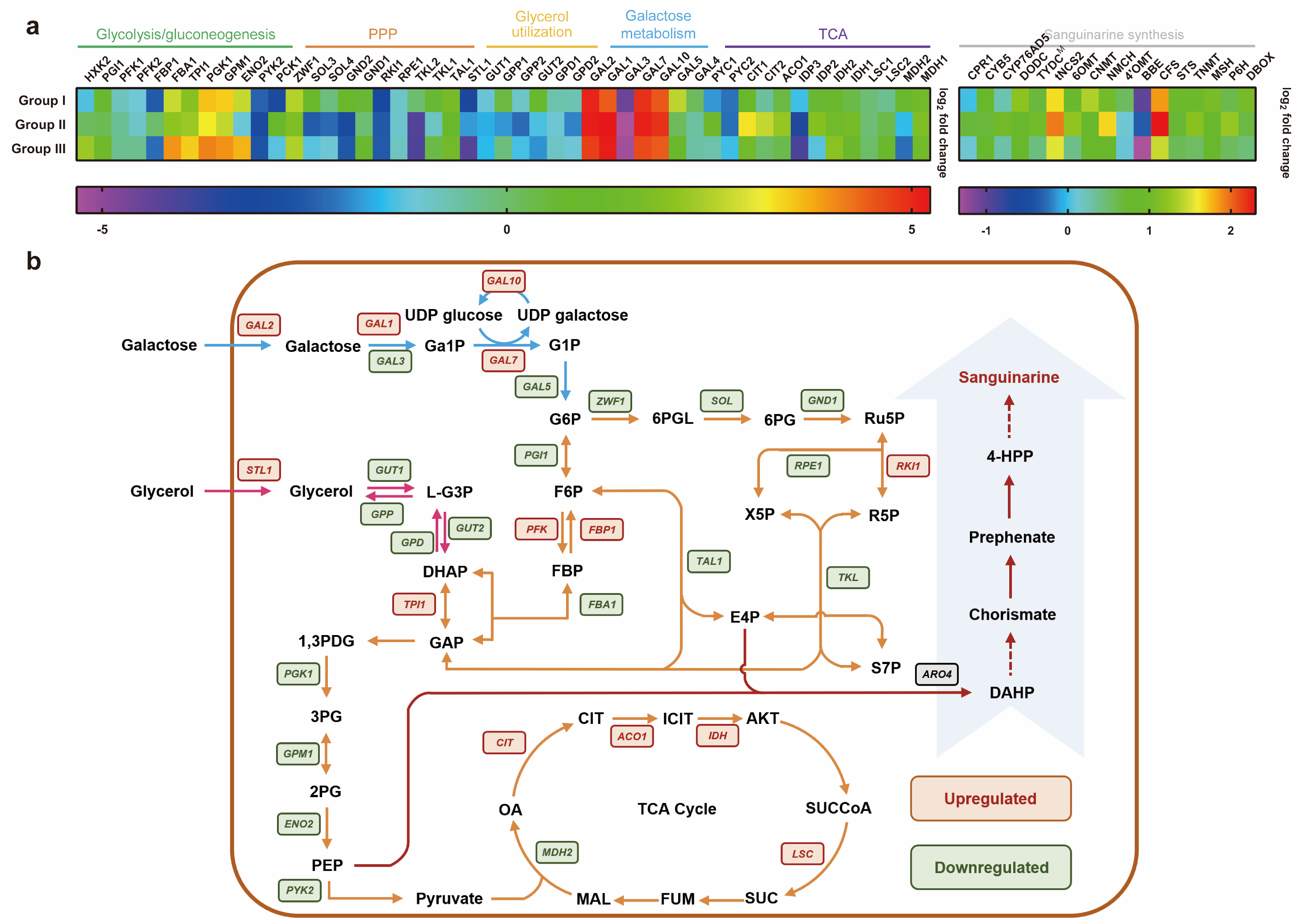


**Supplementary Fig. 17** DEGs related to central carbon metabolism and the sanguinarine synthesis heterologous pathway. (a) Cluster analysis of DEGs associated with glycolysis, the PPP, glycerol utilization, galactose metabolism, the TCA cycle, and sanguinarine synthesis under different carbon source conditions. Group I represents glycerol as the carbon source, Group II represents a combination of glycerol and galactose as the carbon source, and Group III represents galactose as the carbon source. (b) Schematic representation of DEGs within central carbon metabolic pathways when glycerol and galactose are carbon sources, with upregulated genes in red and downregulated genes in green. Data are presented as mean ± s.d. (n = 3 biologically independent samples). Source data are provided as a Source Data file.


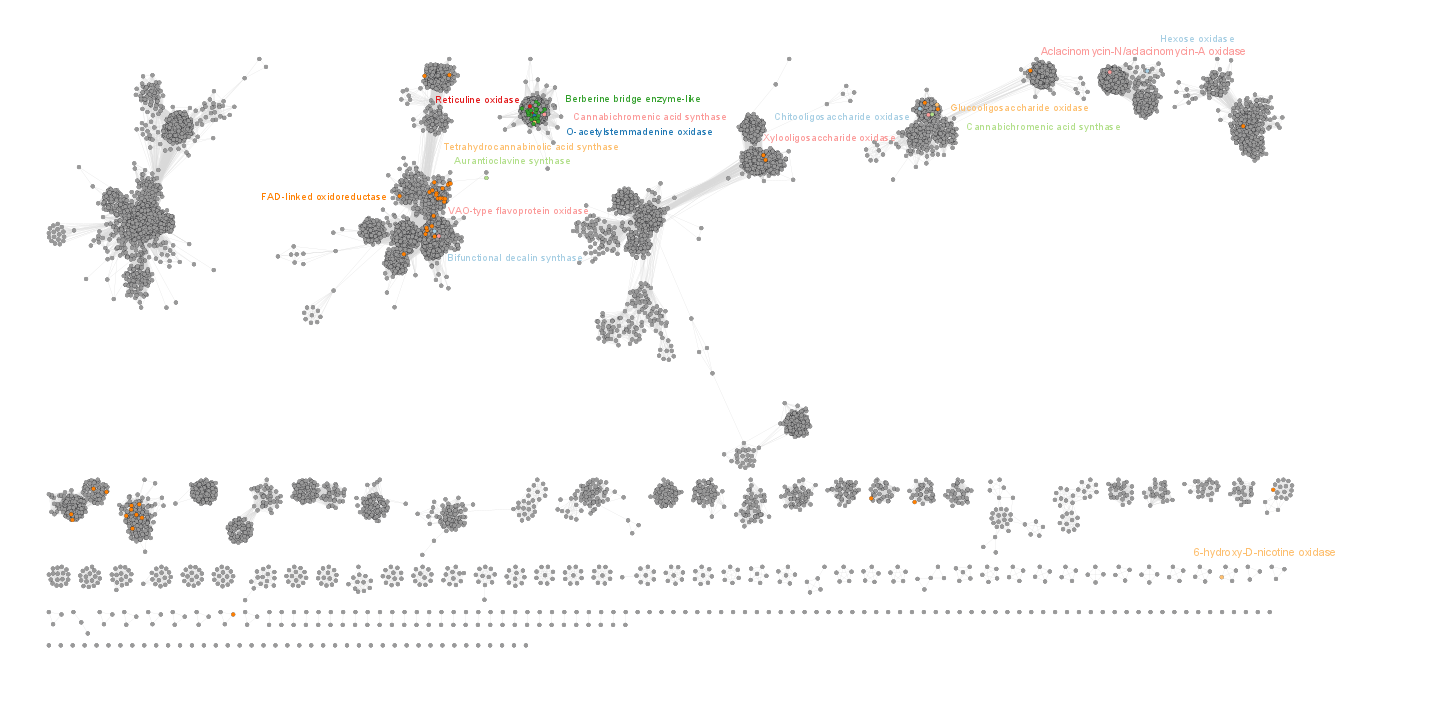
**Supplementary Fig. 18** Sequence Similarity Network (SSN) of the berberine bridge enzyme family (Pfam08031). Using an alignment score threshold (AST) of 94 and restricting sequence lengths to 450-660 amino acids, the figure displays 8,812 sequences containing annotations of 15 different SwissProt descriptions.


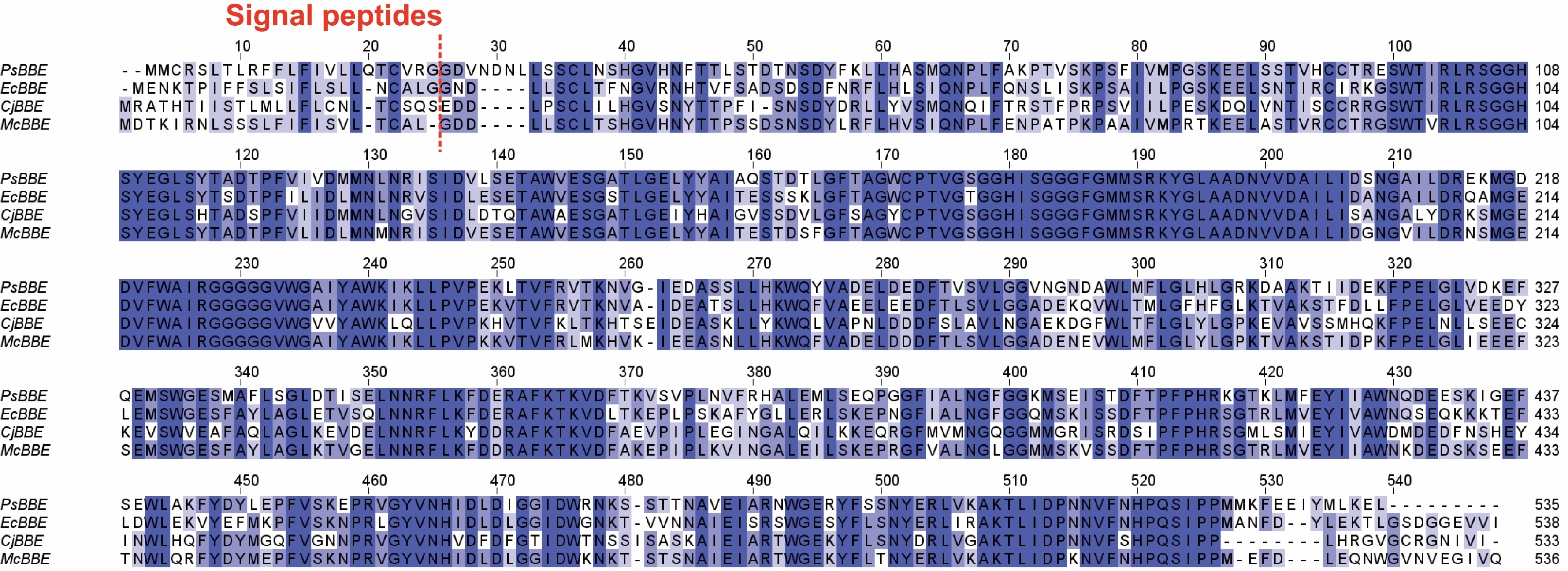
**Supplementary Fig. 19** Protein sequence comparison of reticuline oxidases from four different species and the predicted signal peptides.


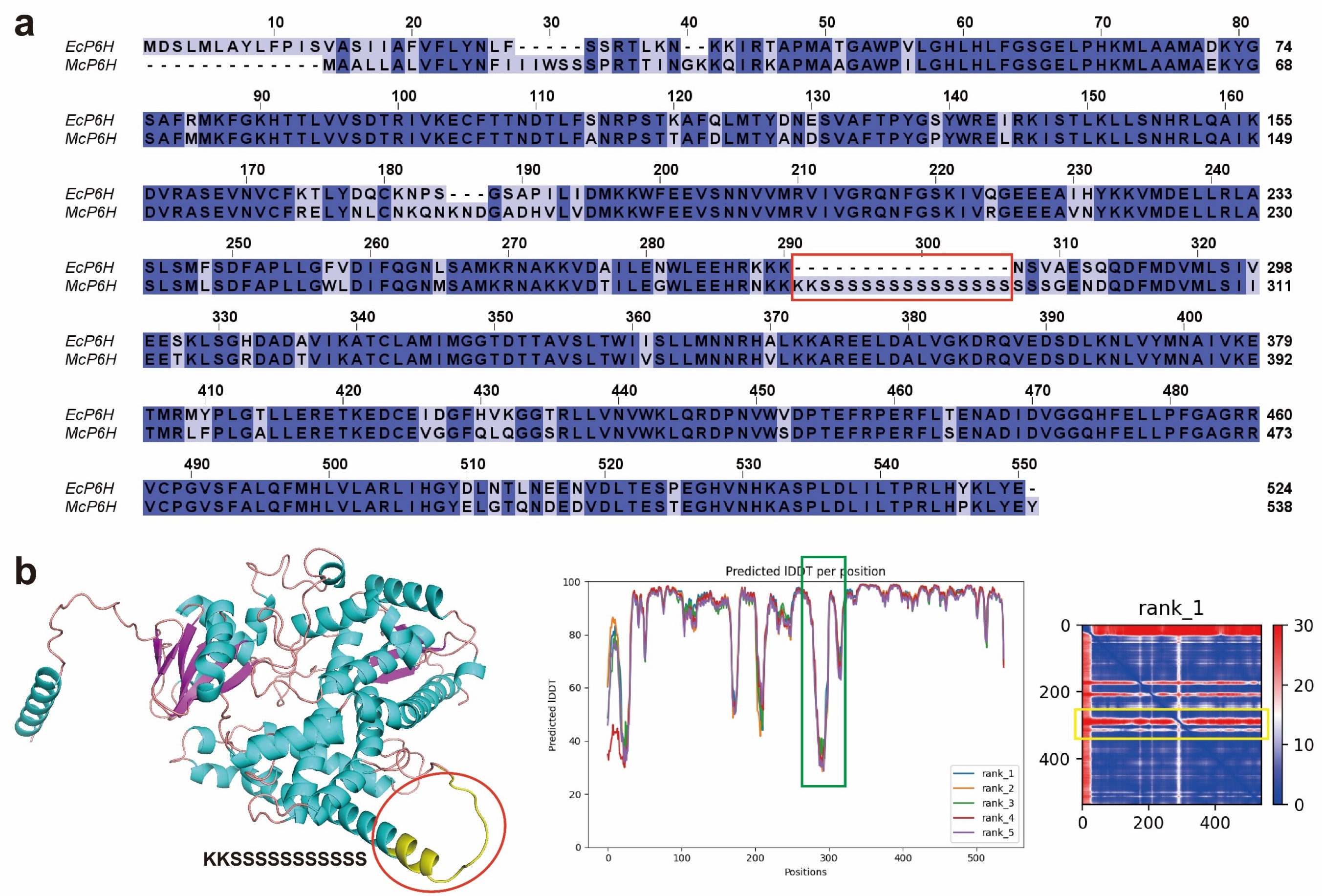
**Supplementary Fig. 20** Protein sequence and structural analysis of protopine 6-hydroxylase (P6H). (a) Protein sequence comparison between *Mc*P6H and *Ec*P6H. (b) Prediction of *Mc*P6H protein structure using AlphaFold 2.0, indicating a low confidence score for consecutive serine residues KKSSSSSSSSSSSSSS.


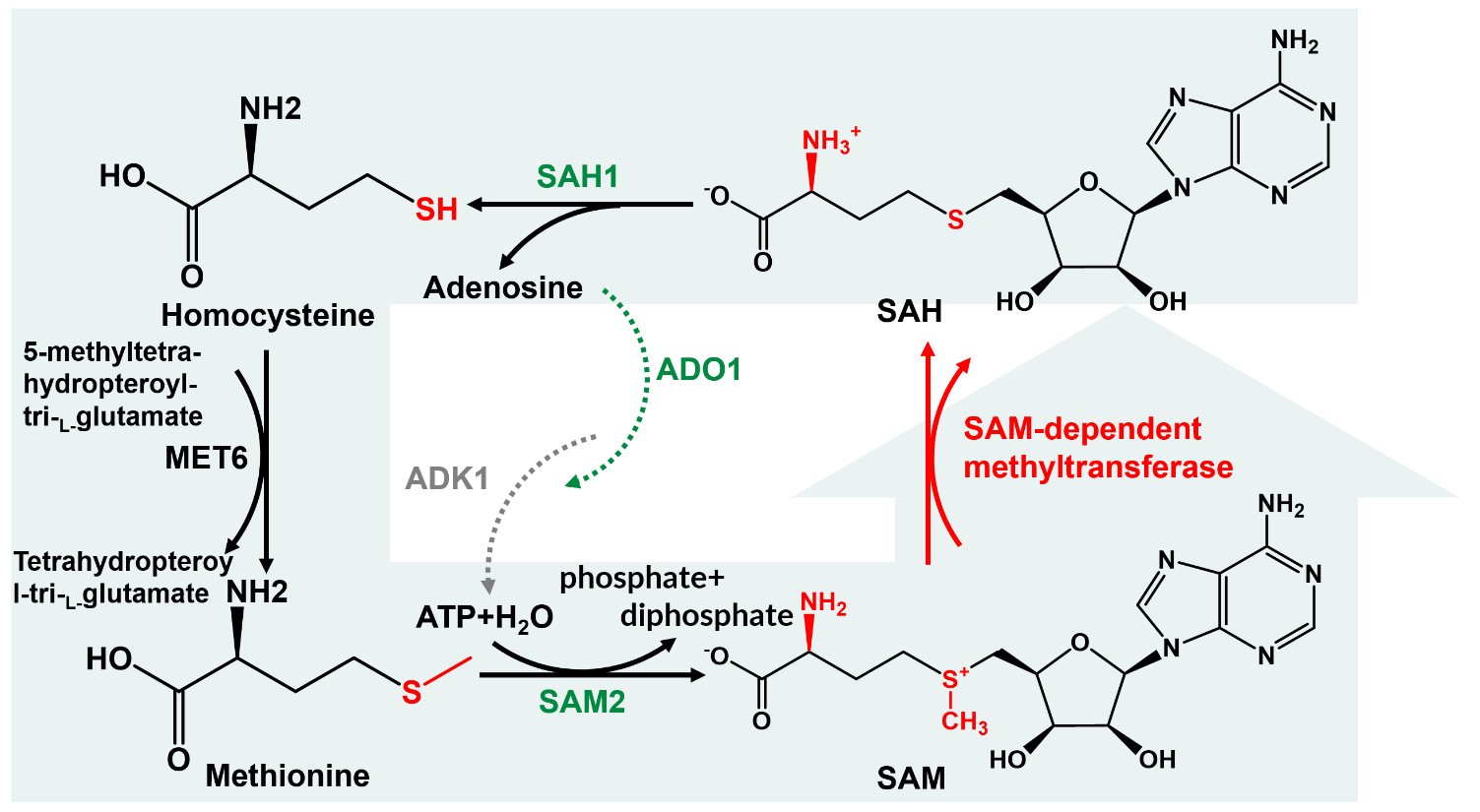
**Supplementary Fig. 21** Schematic illustration of the SAM cycle in yeast**.**


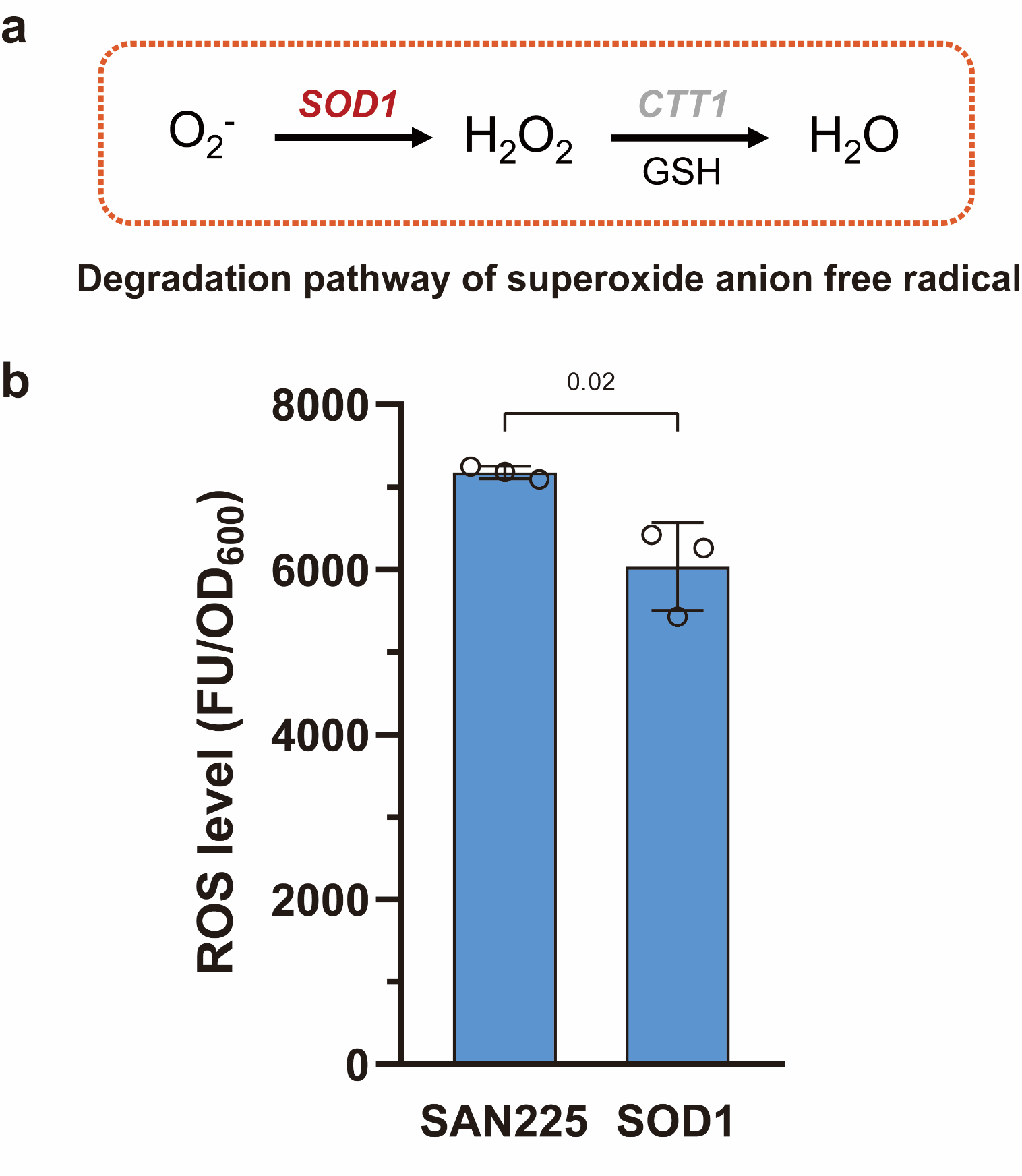
**Supplementary Fig. 22** Improved cell microenvironment with *SOD1* overexpression to decrease ROS level. (a) Schematic illustration of the degradation pathway of the superoxide anion free radical. (b) Intracellular ROS levels pre- and post-overexpression of the *SOD1* gene. Data are presented as mean ± s.d. (n = 3 biologically independent samples). Significance was calculated using the unpaired Student’s t test (two-sided). Source data are provided as a Source Data file.


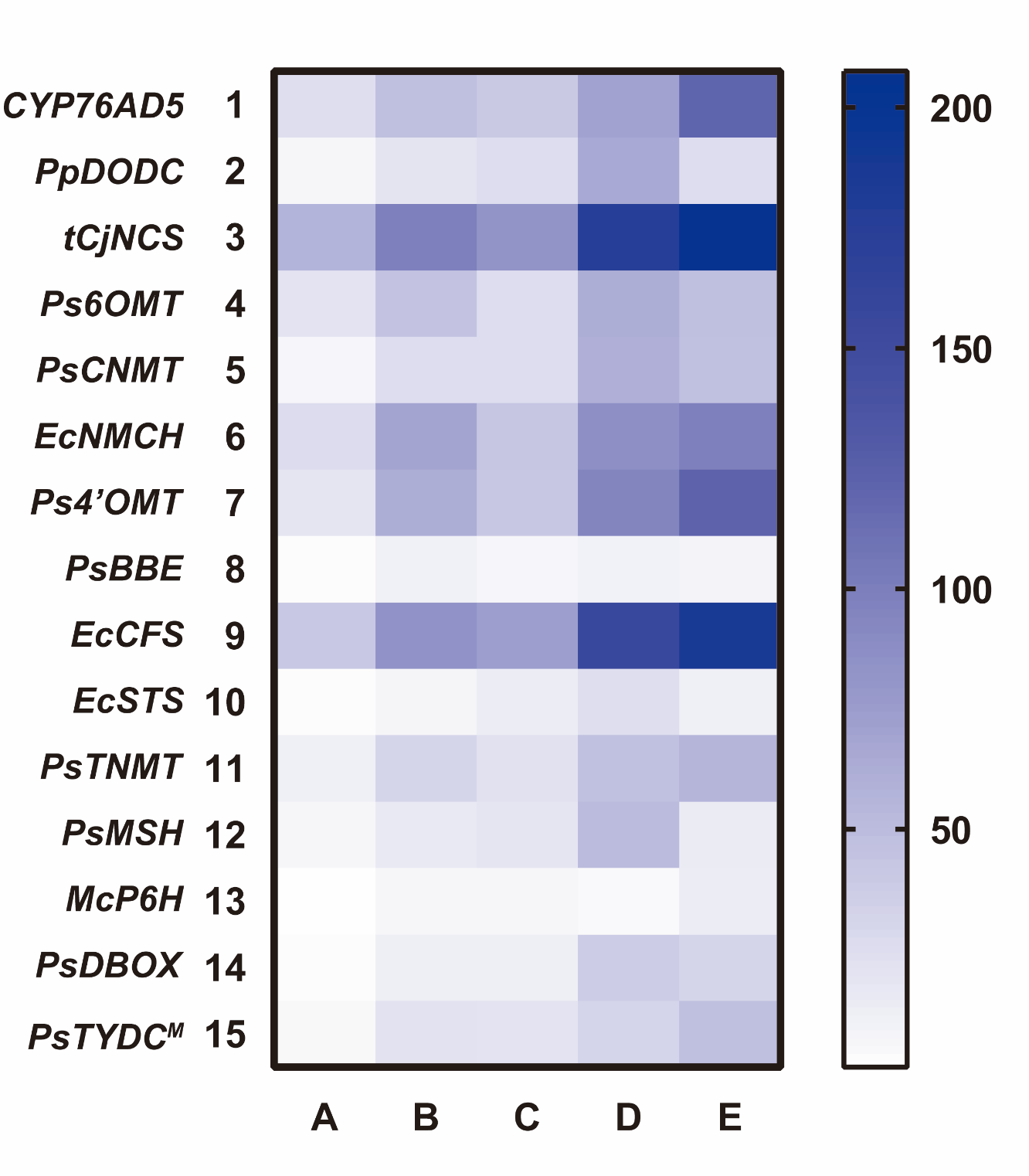
**Supplementary Fig. 23** Relative expression levels of exogenous genes in the SAN220-tsINT strain detected by qPCR. Samples were collected at 12 h, 24 h, and 36 h for each of the five carbon sources, and the samples with the highest expression level under each carbon source condition was chosen for horizontal comparison. Group A corresponds to the strain cultured in SED for 36 h, Group B in SEDG for 24 h, Group C in SEG for 24 h, Group D in SEGly for 24 h, and Group E in SEGlyG for 24 h. Total RNA was extracted from the aforementioned strains, subjected to reverse transcription into cDNA, and analyzed for gene expression levels. Data are presented as mean ± s.d. (n = 3 biologically independent samples). Source data are provided as a Source Data file.


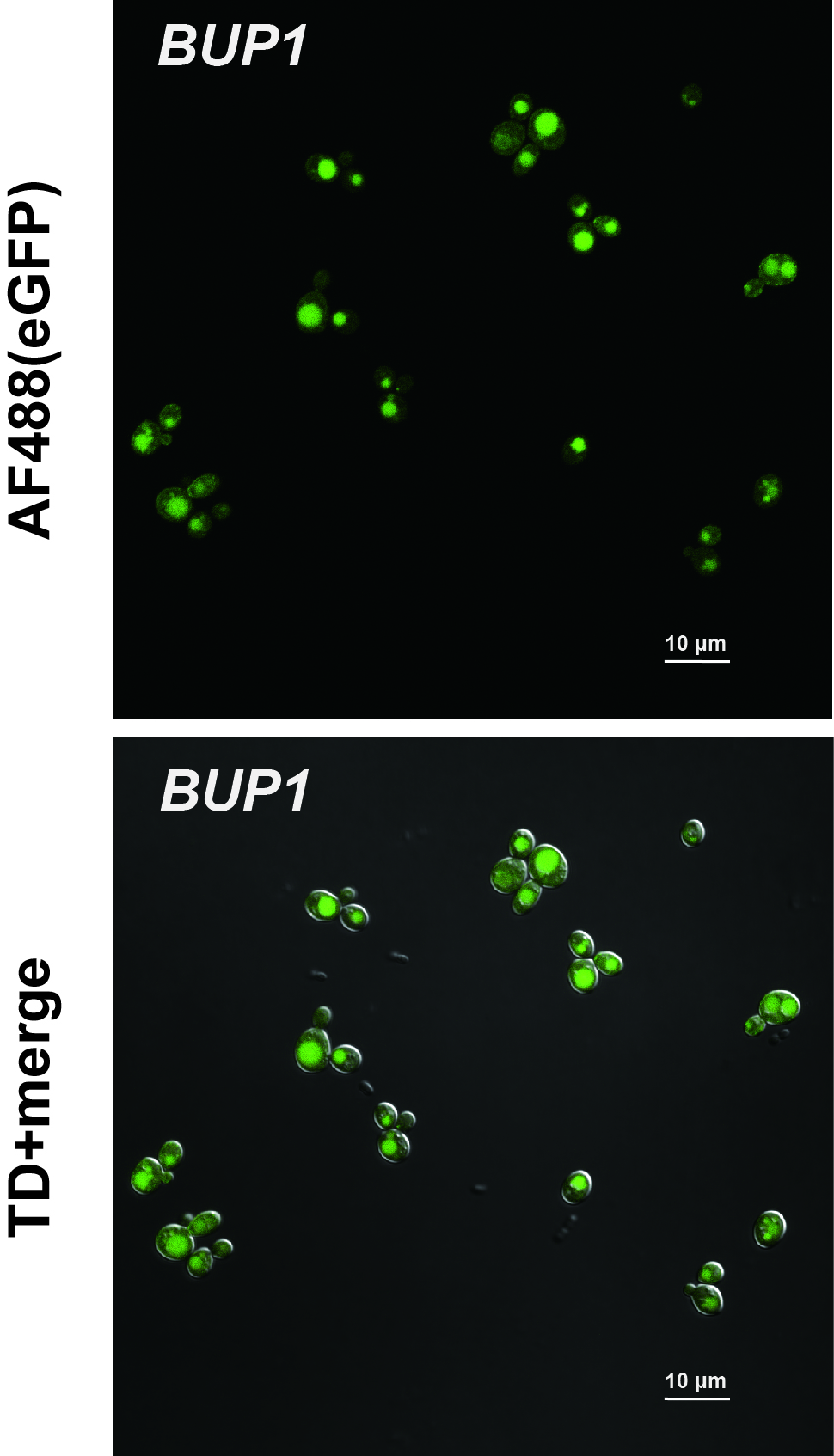
**Supplementary Fig. 24** Subcellular localization of BUP1 by fusing EGFP at the *C-*terminus, observed through fluorescence confocal microscopy. Micrographs were representative of at least two independent experiments.

**
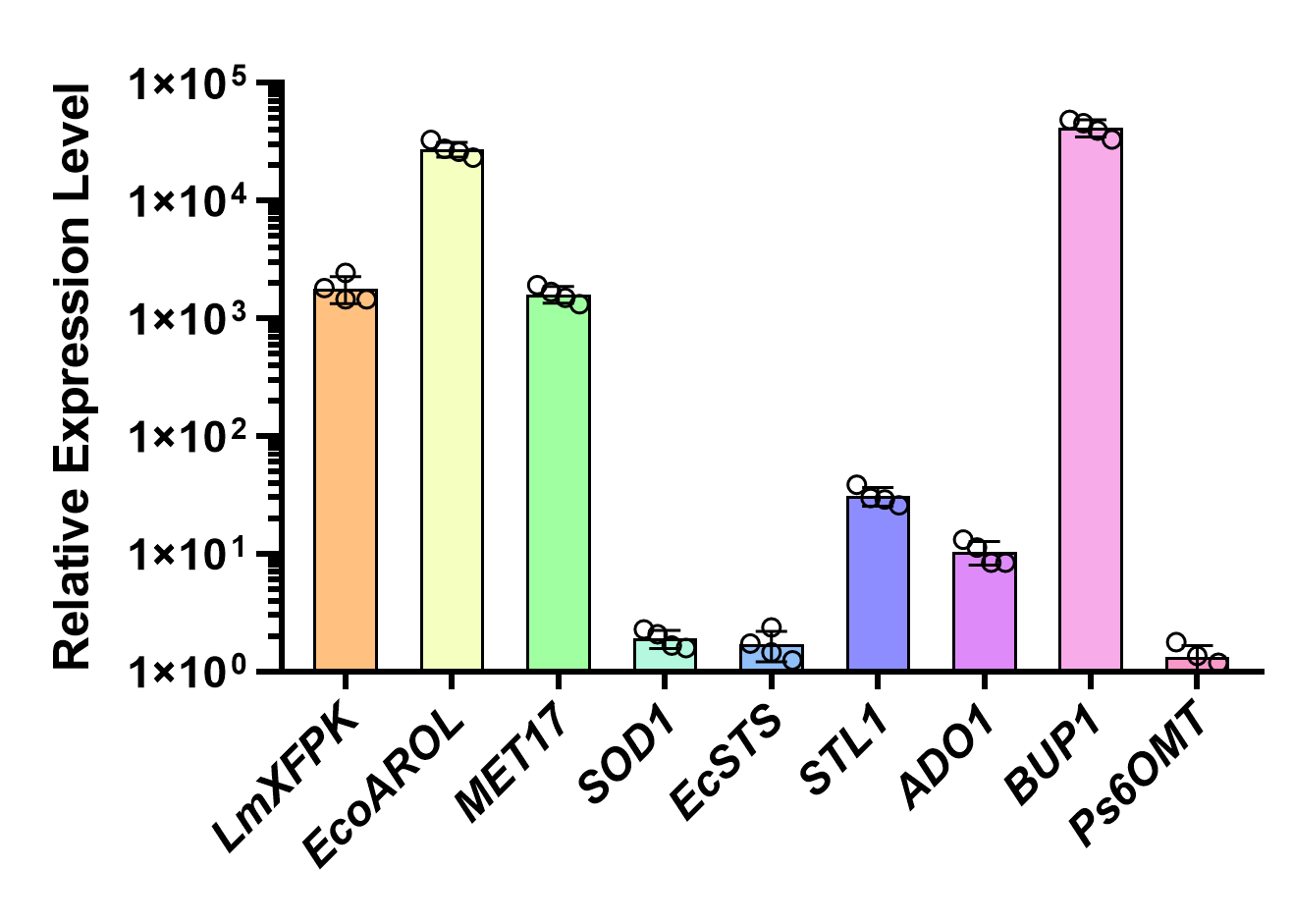
Supplementary Fig. 25** Quantitative analysis of gene expression levels between strains SAN223-4 and SAN231 using qPCR. Data are presented as mean ± s.d. (n = 3 biologically independent samples). Source data are provided as a Source Data file.


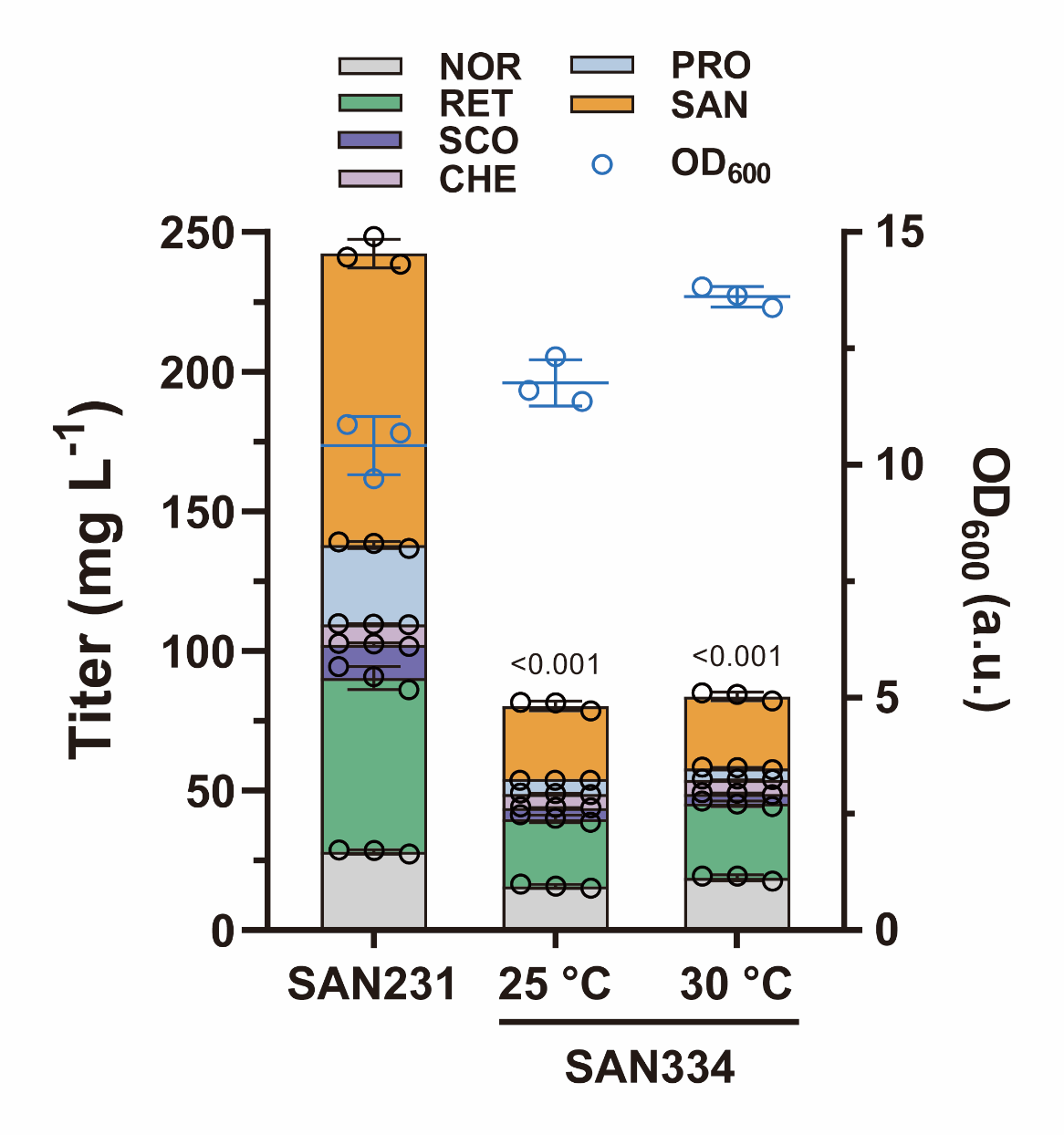
**Supplementary Fig. 26** Validation of SIMTeGES by replacing GAL4-tsINT of strain SAN231 with the wild-type GAL4. Strain SAN334 was cultured in shake flasks at both 25 °C and 30 °C. Abbreviations not previously defined: NOR, (*S*)-Norcoclaurine; RET, (*S*)-Reticuline; SCO, (*S*)-Scoulerine; CHE, (*S*)-Cheilanthifoline; PRO, Protopine; SAN, Sanguinarine. Data are presented as mean ± s.d. (n = 3 biologically independent samples). The significance of sanguinarine titer differences was calculated using two-way ANOVA followed by Tukey’s multiple comparisons test. Source data are provided as a Source Data file.


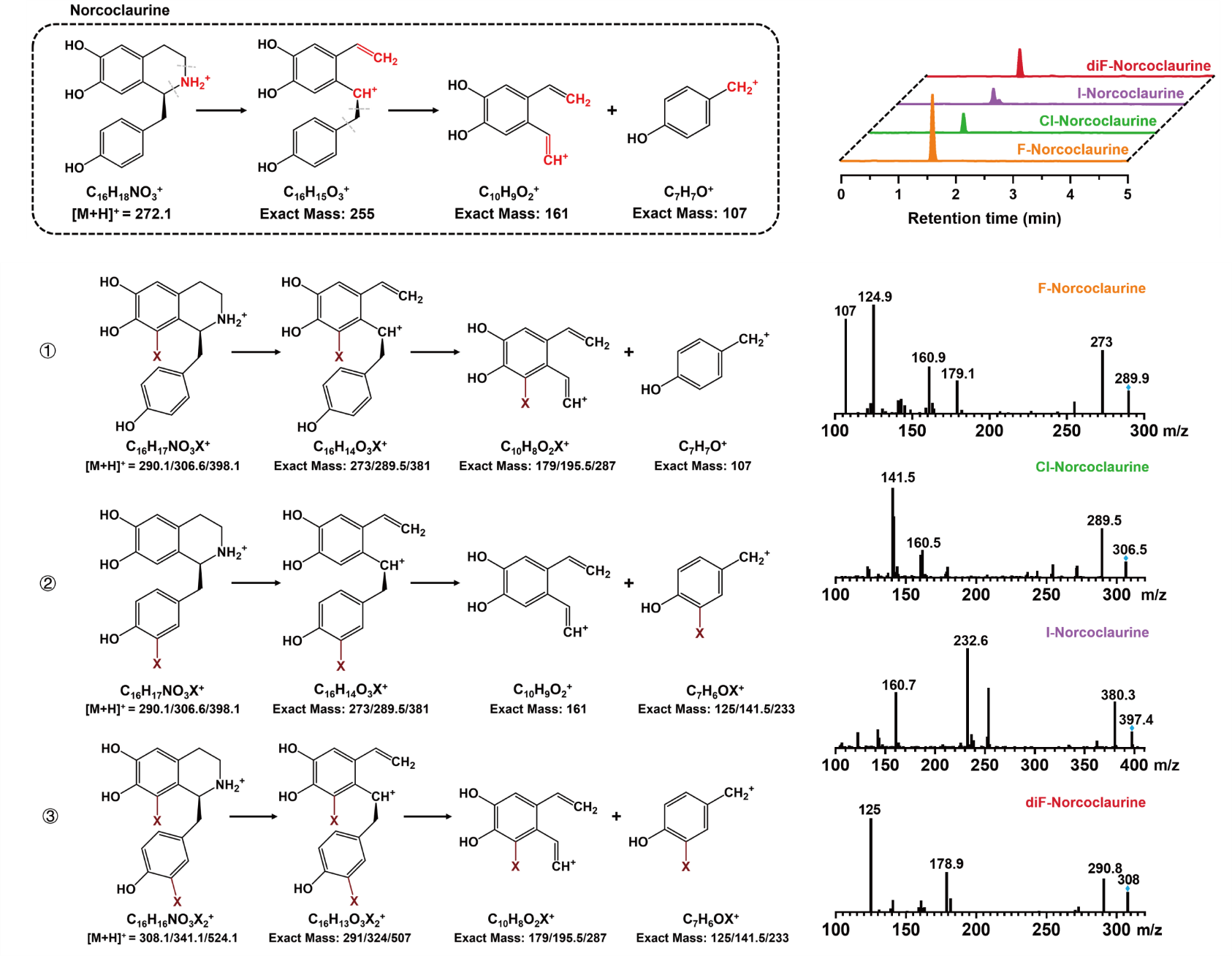


**Supplementary Fig. 27** MS^2^ spectra of F-norcoclaurine, Cl-norcoclaurine, I-norcoclaurine and diF-norcoclaurine detected via production mode (LC-QQQ), displaying potential characteristic fragments and the proportion of halogenations at different positions.


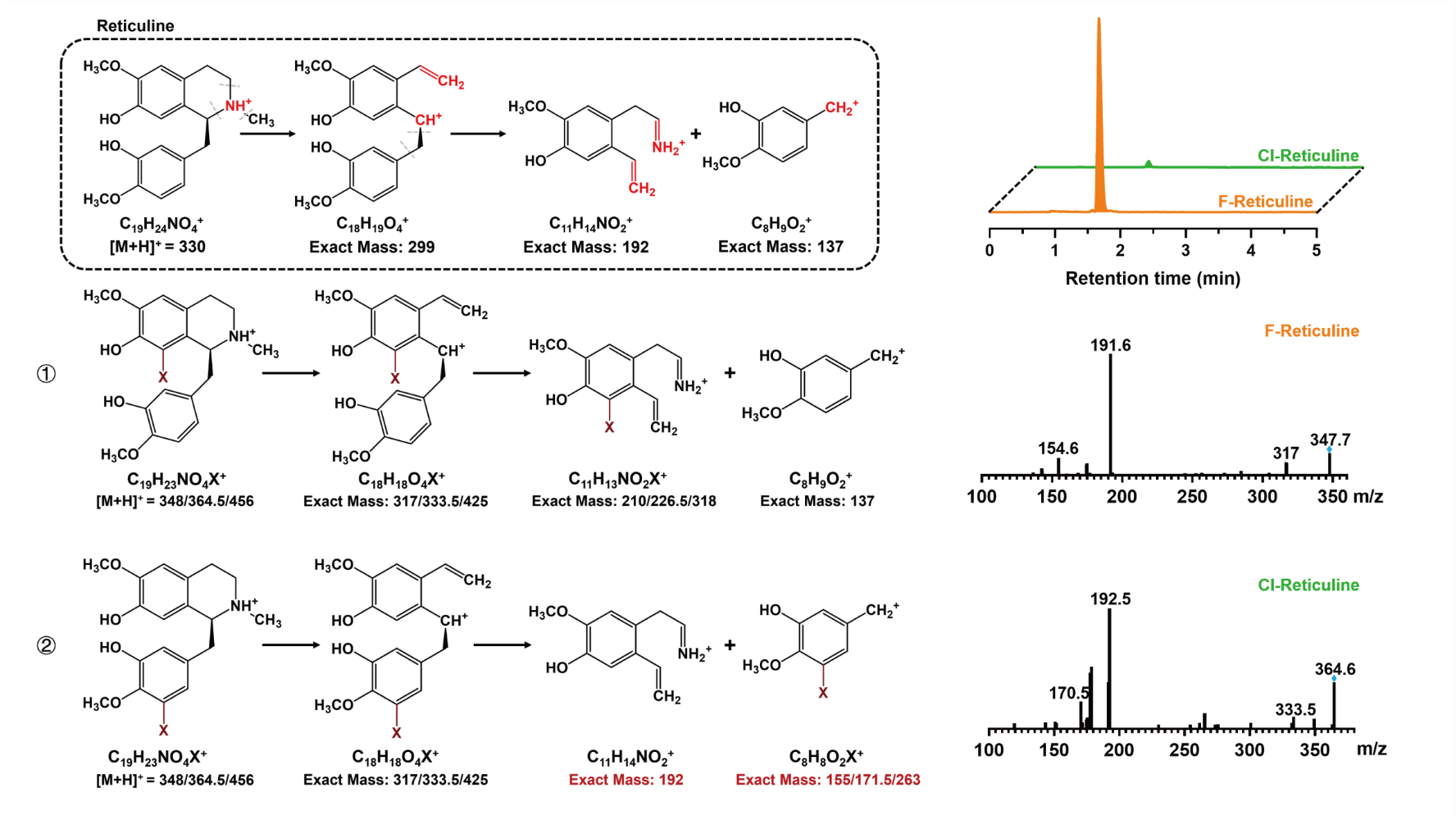


**Supplementary Fig. 28** MS^2^ spectra of Cl-reticuline detected through production mode (LC-QQQ), showcasing potential characteristic fragments and the proportion of halogenations at different positions.


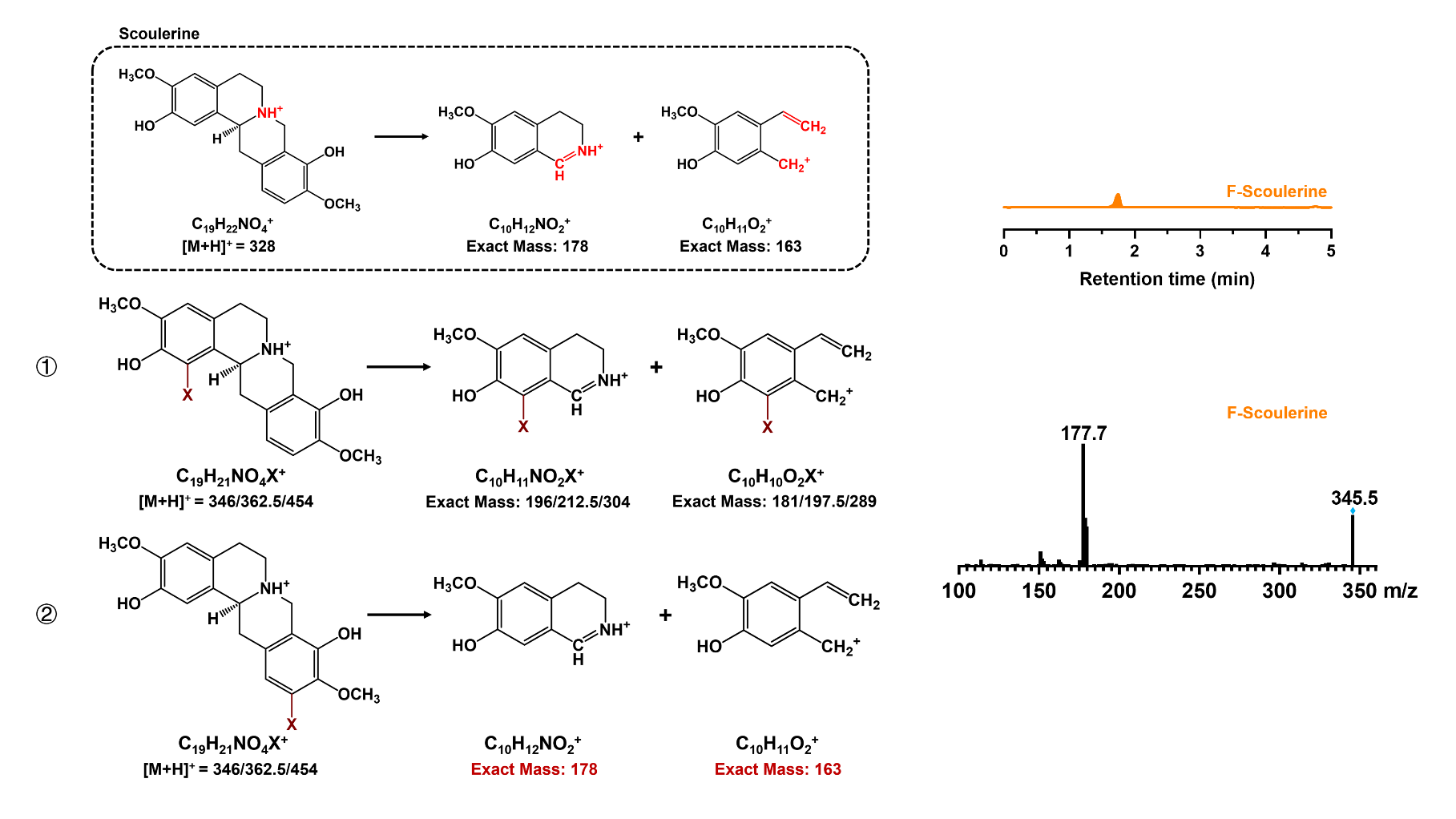


**Supplementary Fig. 29** MS^2^ spectra of F-scoulerine detected through production mode (LC-QQQ), showcasing potential characteristic fragments and the proportion of halogenations at different positions.


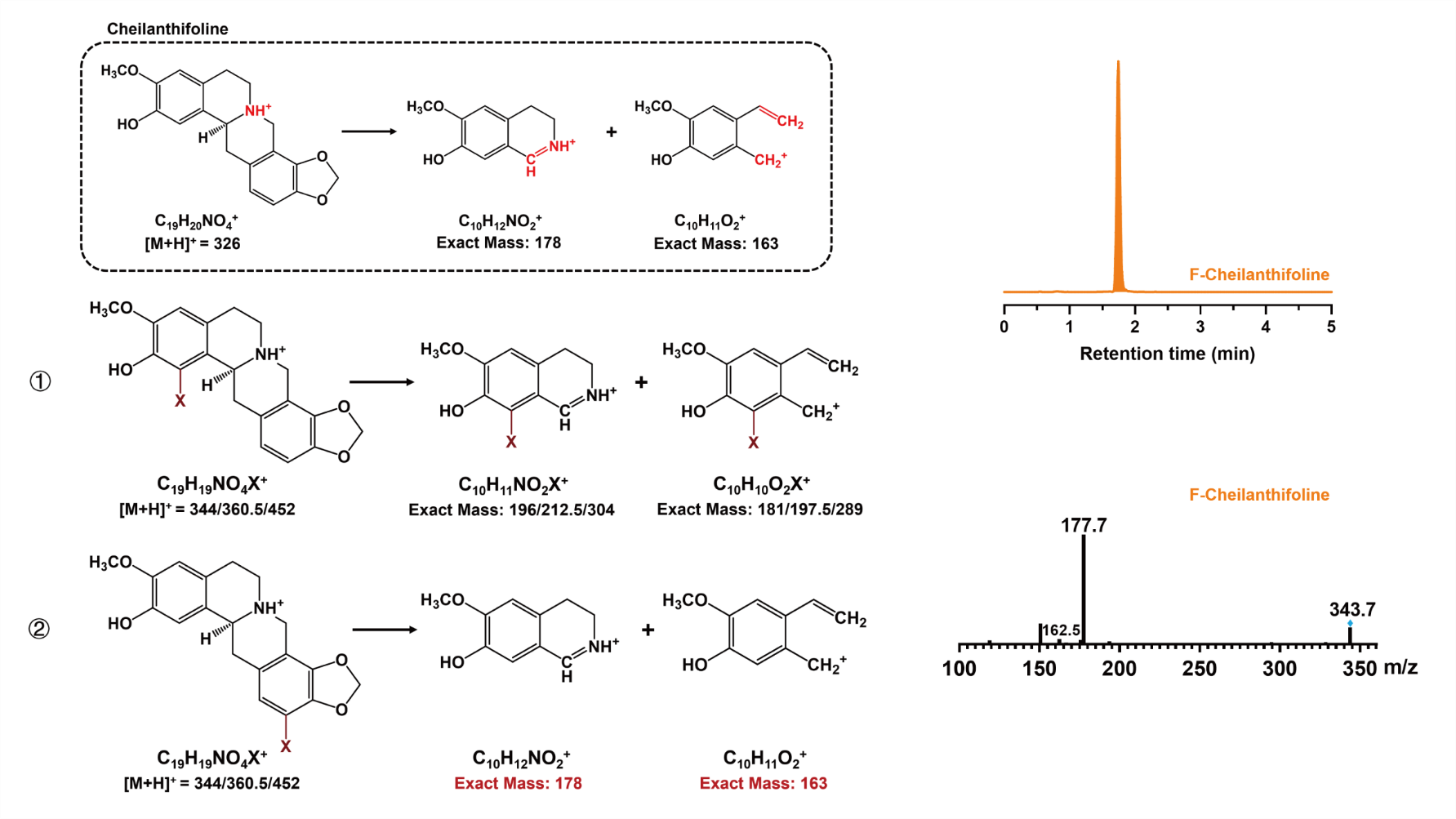


**Supplementary Fig. 30** MS^2^ spectra of F-cheilanthifoline detected through production mode (LC-QQQ), showcasing potential characteristic fragments and the proportion of halogenations at different positions.


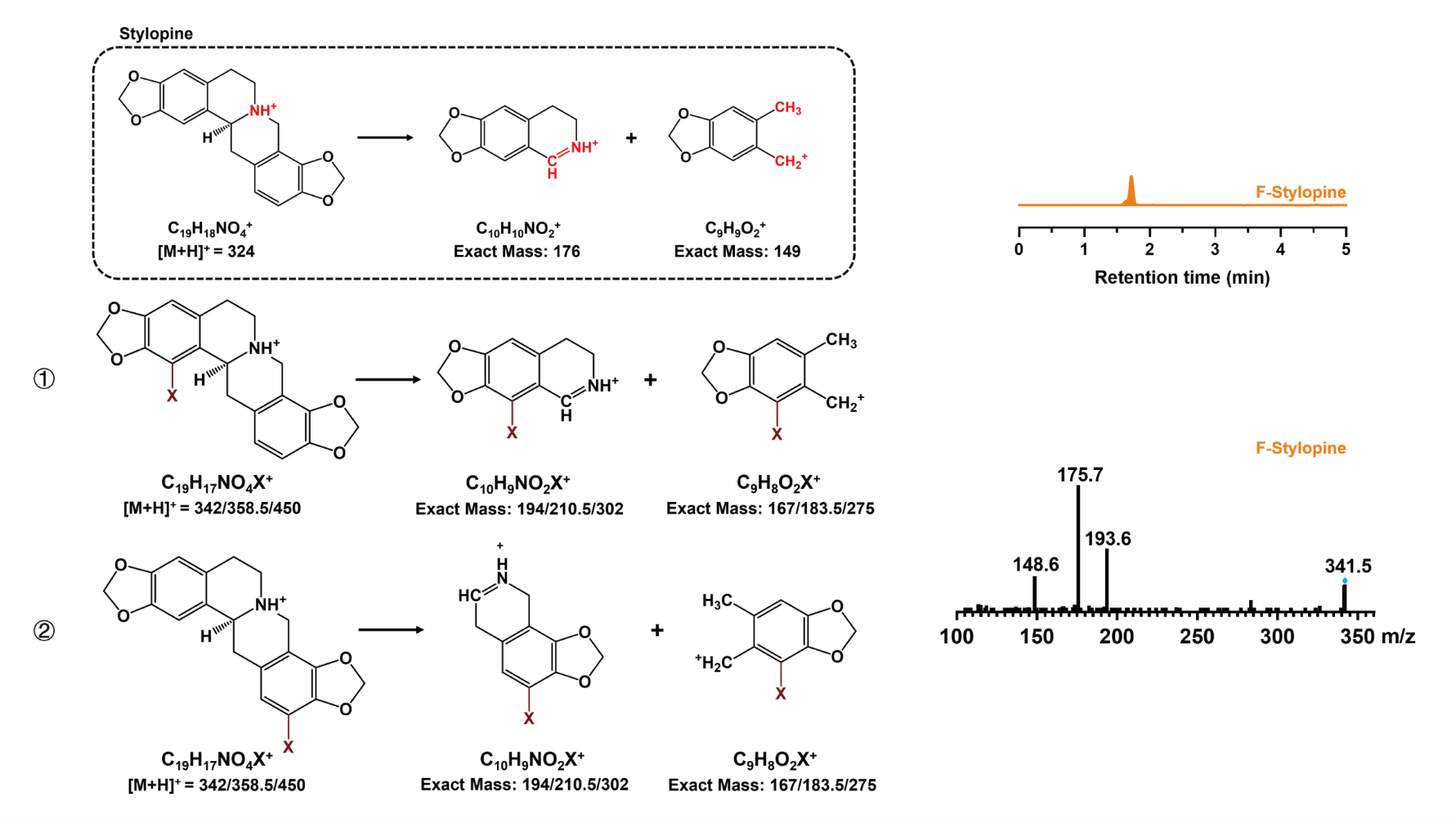


**Supplementary Fig. 31** MS^2^ spectra of F-stylopine detected through production mode (LC-QQQ), showcasing potential characteristic fragments and the proportion of halogenations at different positions.


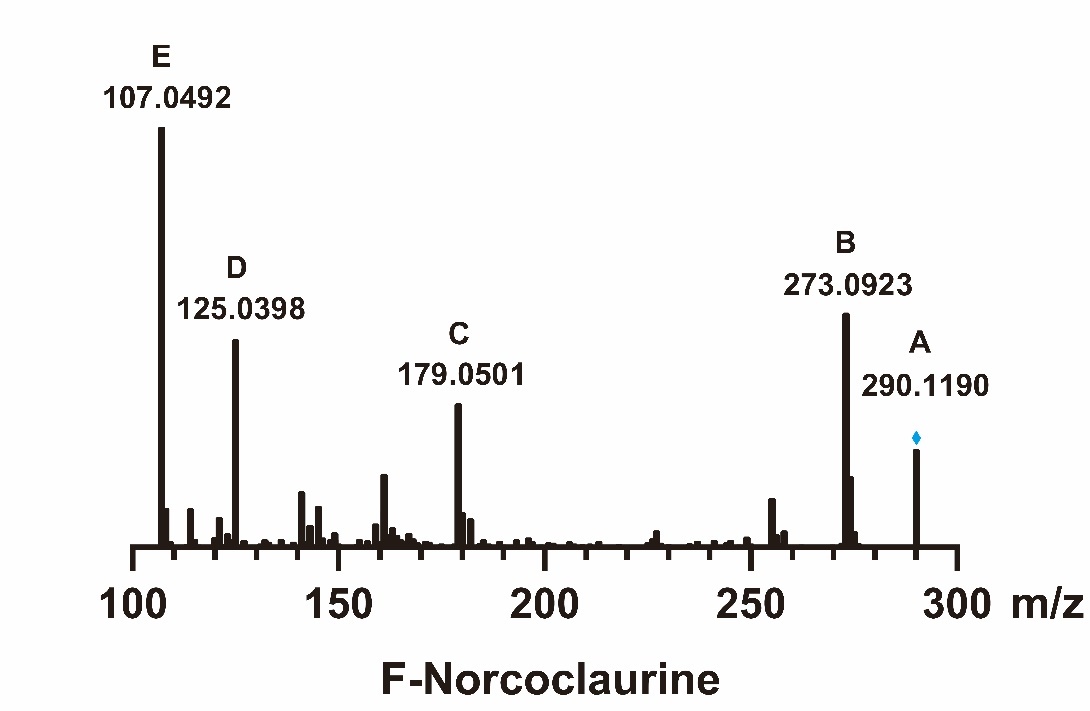


| ***m/z* (observed)** | **Species** | **Predicted formula** | **Ion Assignment** | ***m/z* (theoretical)** | **△*m/z* (ppm)** |
| --- | --- | --- | --- | --- | --- |
| 290.1190 | [M+H]^+^ | C_16_H_17_FNO_3_ | A | 290.1187 | 1.94 |
| 273.0923 | [M+H]^+^ | C_16_H_14_FO_3_ | B | 273.0921 | 1.16 |
| 179.0501 | [M+H]^+^ | C_10_H_8_FO_2_ | C | 179.0503 | -0.83 |
| 125.0398 | [M+H]^+^ | C_7_H_6_FO | D | 125.0397 | 0.61 |
| 107.0492 | [M+H]^+^ | C_7_H_7_O | E | 107.0491 | -0.15 |

**Supplementary Fig. 32** High-resolution MS^2^ spectra of F-norcoclaurine, displaying potential fragment formulas. Source data are provided as a Source Data file.


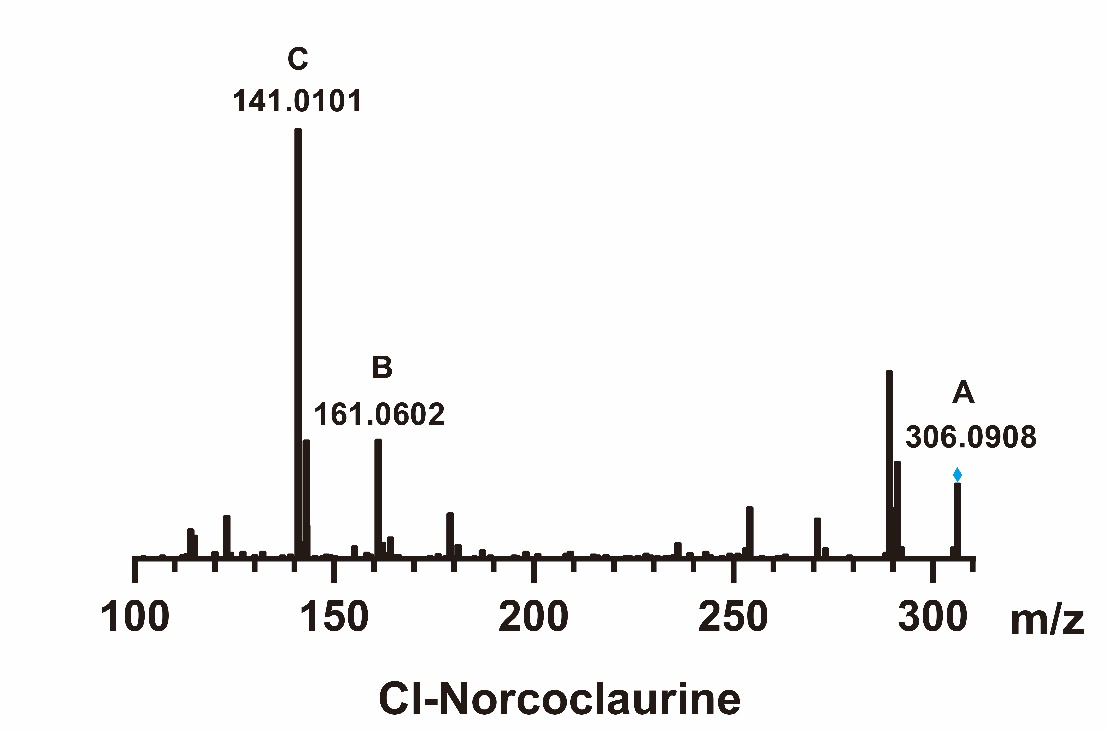


| ***m/z* (observed)** | **Species** | **Predicted formula** | **Ion Assignment** | ***m/z* (theoretical)** | **△*m/z* (ppm)** |
| --- | --- | --- | --- | --- | --- |
| 306.0908 | [M+H]^+^ | C_16_H_17_ClNO_3_ | A | 306.0891 | 5.91 |
| 161.0602 | [M+H]^+^ | C_10_H_9_O_2_ | B | 161.0597 | 2.30 |
| 141.0101 | [M+H]^+^ | C_7_H_6_ClO | C | 141.0102 | -0.27 |

**Supplementary Fig. 33** High-resolution MS^2^ spectra of Cl-norcoclaurine, displaying potential fragment formulas. Source data are provided as a Source Data file.


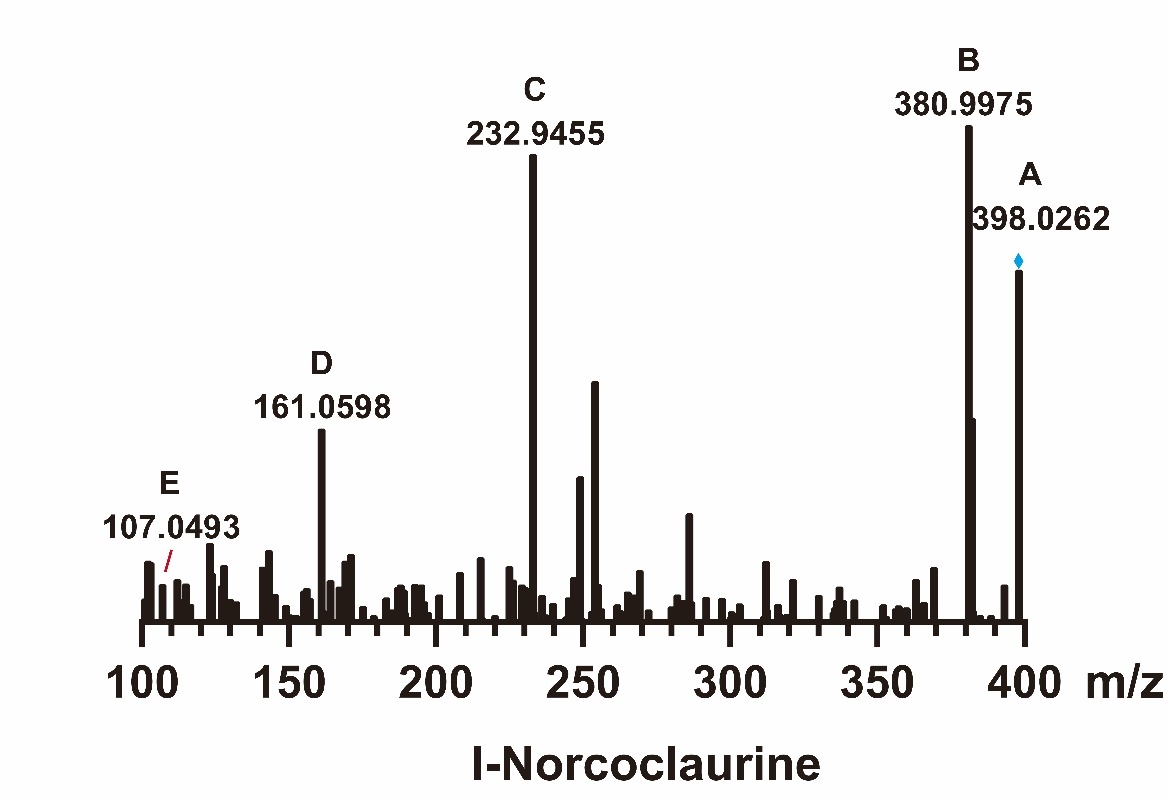


| ***m/z* (observed)** | **Species** | **Predicted formula** | **Ion Assignment** | ***m/z* (theoretical)** | **△*m/z* (ppm)** |
| --- | --- | --- | --- | --- | --- |
| 398.0262 | [M+H]^+^ | C_16_H_17_INO_3_ | A | 398.0248 | 2.19 |
| 380.9975 | [M+H]^+^ | C_16_H_14_IO_3_ | B | 380.9982 | 3.00 |
| 232.9455 | [M+H]^+^ | C_7_H_6_IO | C | 232.9458 | -1.17 |
| 161.0598 | [M+H]^+^ | C_10_H_9_O_2_ | D | 161.0597 | 15.36 |
| 107.0493 | [M+H]^+^ | C_7_H_7_O | E | 107.0491 | 1.61 |

**Supplementary Fig. 34** High-resolution MS^2^ spectra of I-norcoclaurine, displaying potential fragment formulas. Source data are provided as a Source Data file.


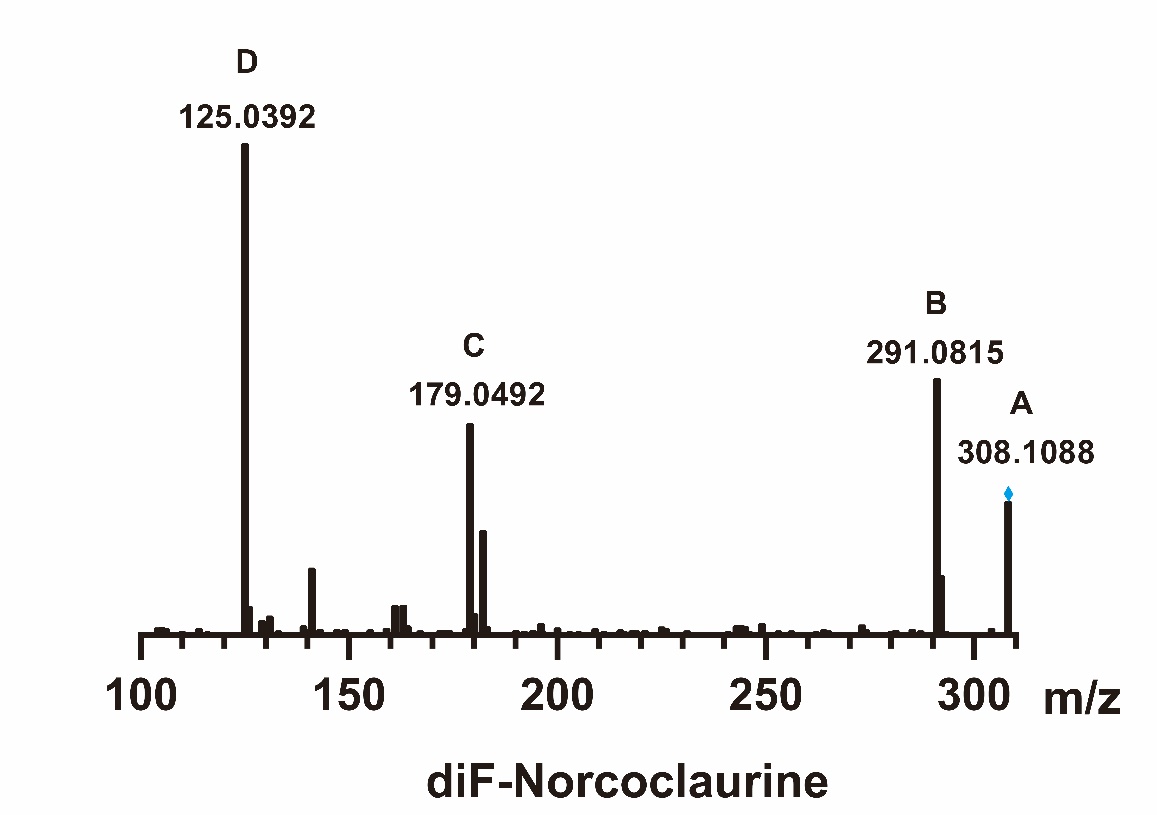


| ***m/z* (observed)** | **Species** | **Predicted formula** | **Ion Assignment** | ***m/z* (theoretical)** | **△*m/z* (ppm)** |
| --- | --- | --- | --- | --- | --- |
| 308.1088 | [M+H]^+^ | C_16_H_16_F_2_NO_3_ | A | 308.1093 | -2.43 |
| 291.0815 | [M+H]^+^ | C_16_H_13_F_2_O_3_ | B | 291.0827 | -4.01 |
| 179.0492 | [M+H]^+^ | C_10_H_8_FO_2_ | C | 179.0503 | -6.38 |
| 125.0392 | [M+H]^+^ | C_7_H_6_FO | D | 125.0397 | -4.1 |

**Supplementary Fig. 35** High-resolution MS^2^ spectra of diF-norcoclaurine, displaying potential fragment formulas. Source data are provided as a Source Data file.


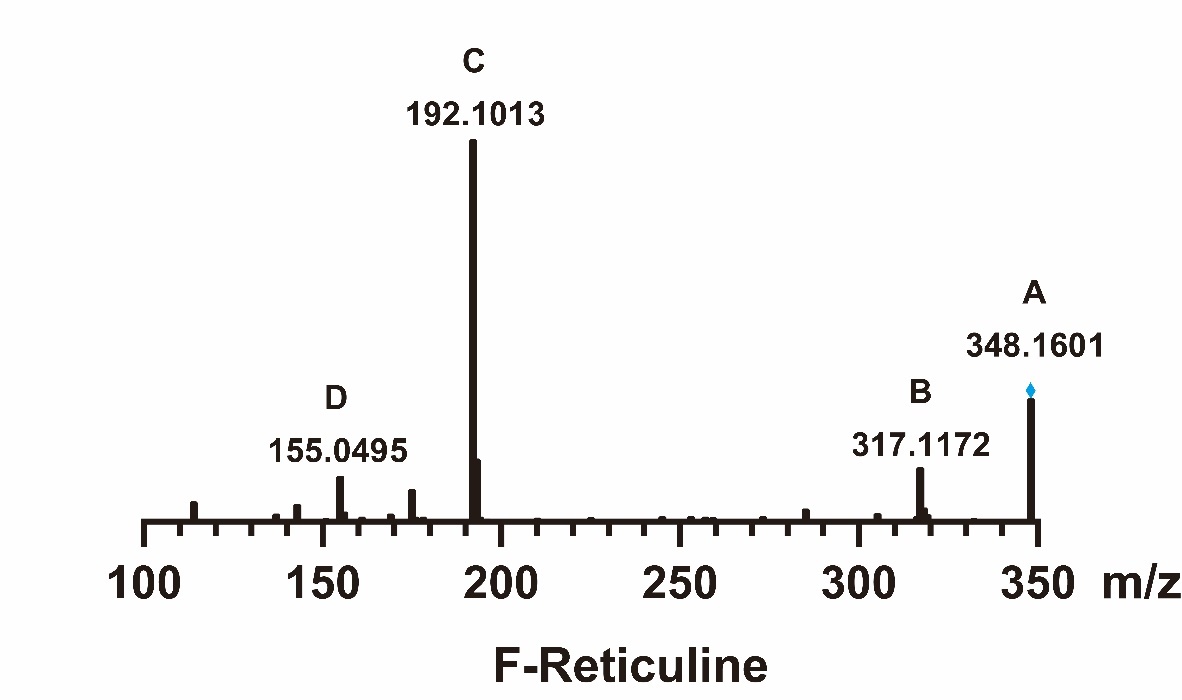


| ***m/z* (observed)** | **Species** | **Predicted formula** | **Ion Assignment** | ***m/z* (theoretical)** | **△*m/z* (ppm)** |
| --- | --- | --- | --- | --- | --- |
| 348.1601 | [M+H]^+^ | C_19_H_23_FNO_4_ | A | 348.1606 | -1.21 |
| 317.1172 | [M+H]^+^ | C_18_H_18_FO_4_ | B | 317.1184 | -3.32 |
| 192.1013 | [M+H]^+^ | C_11_H_14_NO_2_ | C | 192.1019 | -2.78 |
| 155.0495 | [M+H]^+^ | C_8_H_8_FO_2_ | D | 155.0503 | -5.11 |

**Supplementary Fig. 36** High-resolution MS^2^ spectra of F-reticuline, displaying potential fragment formulas. Source data are provided as a Source Data file.


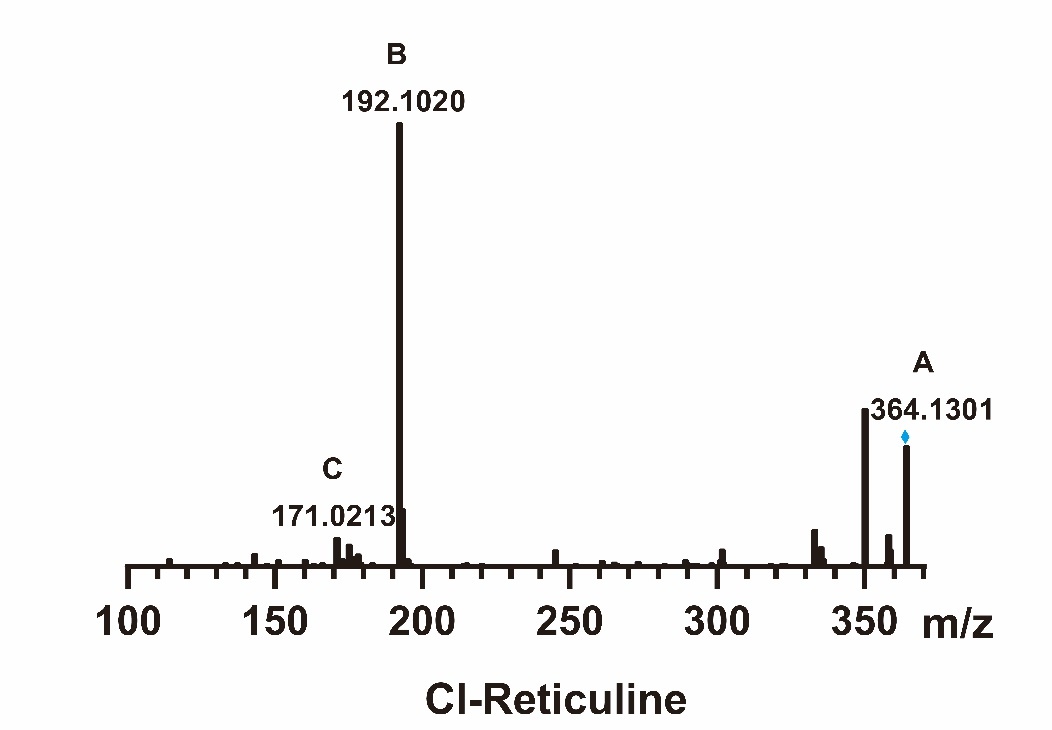


| ***m/z* (observed)** | **Species** | **Predicted formula** | **Ion Assignment** | ***m/z* (theoretical)** | **△*m/z* (ppm)** |
| --- | --- | --- | --- | --- | --- |
| 364.1301 | [M+H]^+^ | C_19_H_23_ClNO_4_ | A | 364.1310 | -2.86 |
| 192.1020 | [M+H]^+^ | C_11_H_14_NO_2_ | B | 192.1019 | 0.58 |
| 171.0213 | [M+H]^+^ | C_8_H_8_ClO_2_ | C | 171.0207 | -2.67 |

**Supplementary Fig. 37** High-resolution MS^2^ spectra of Cl-reticuline, displaying potential fragment formulas. Source data are provided as a Source Data file.


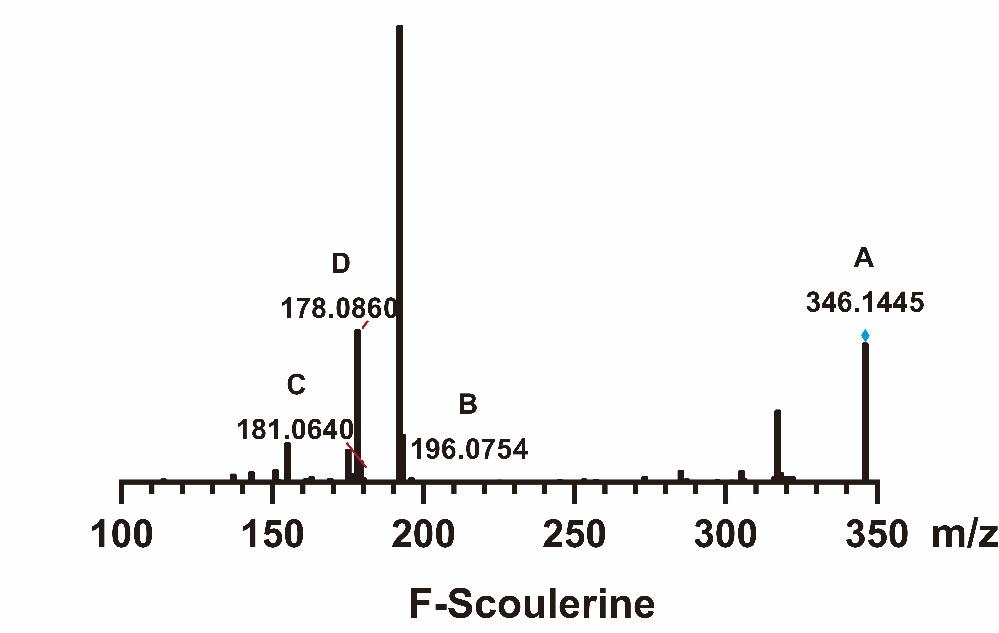


| ***m/z* (observed)** | **Species** | **Predicted formula** | **Ion Assignment** | ***m/z* (theoretical)** | **△*m/z* (ppm)** |
| --- | --- | --- | --- | --- | --- |
| 346.1445 | [M+H]^+^ | C_19_H_21_FNO_4_ | A | 346.1449 | -0.71 |
| 196.0754 | [M+H]^+^ | C_10_H_11_FNO_2_ | B | 196.0768 | -6.76 |
| 181.0640 | [M+H]^+^ | C_10_H_10_FO_2_ | C | 181.0659 | -10.58 |
| 178.0860 | [M+H]^+^ | C_10_H_12_NO_2_ | D | 178.0863 | -0.74 |

**Supplementary Fig. 38** High-resolution MS^2^ spectra of F-scoulerine, displaying potential fragment formulas. Source data are provided as a Source Data file.


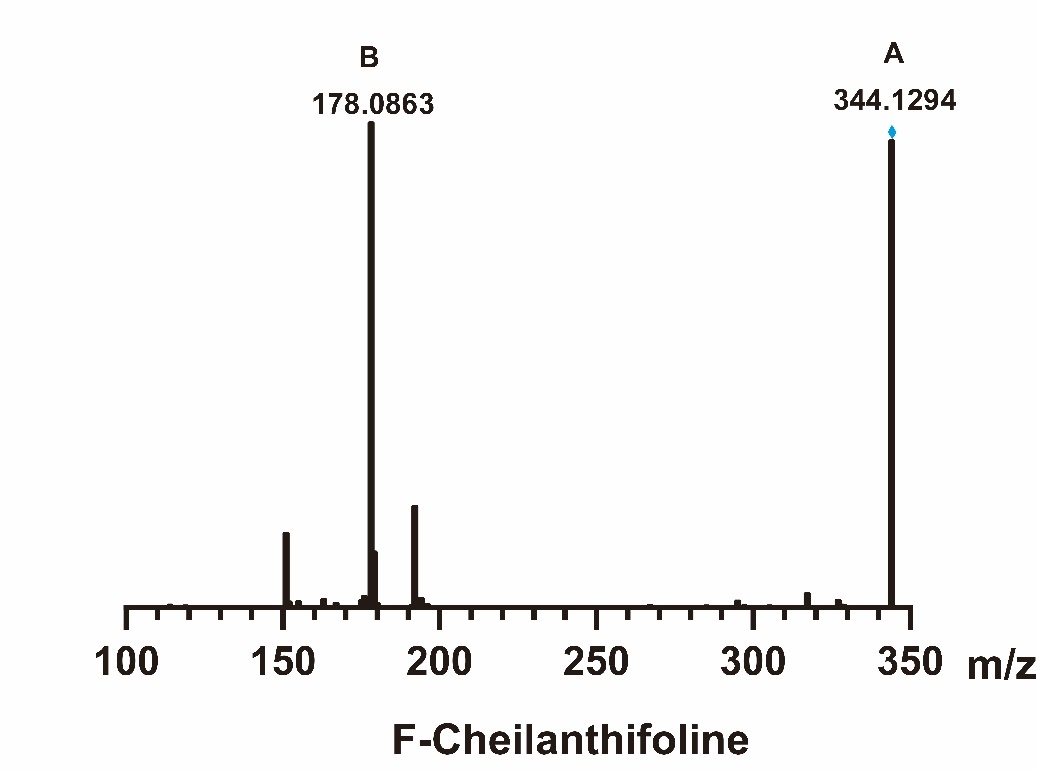


| ***m/z* (observed)** | **Species** | **Predicted formula** | **Ion Assignment** | ***m/z* (theoretical)** | **△*m/z* (ppm)** |
| --- | --- | --- | --- | --- | --- |
| 344.1294 | [M+H]^+^ | C_19_H_19_FNO_4_ | A | 344.1293 | 0.26 |
| 178.0863 | [M+H]^+^ | C_10_H_12_NO_2_ | B | 178.0863 | 0.01 |

**Supplementary Fig. 39** High-resolution MS^2^ spectra of F-cheilanthifoline, displaying potential fragment formulas. Source data are provided as a Source Data file.


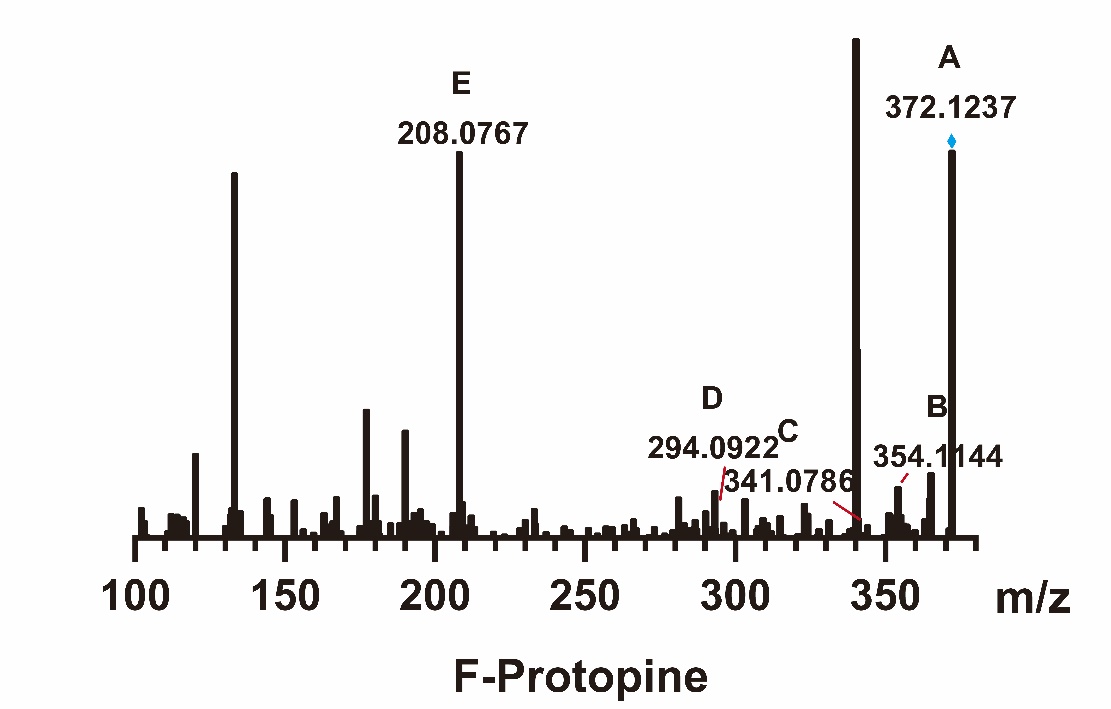


| ***m/z* (observed)** | **Species** | **Predicted formula** | **Ion Assignment** | ***m/z* (theoretical)** | **△*m/z* (ppm)** |
| --- | --- | --- | --- | --- | --- |
| 372.1237 | [M+H]^+^ | C_20_H_19_FNO_5_ | A | 372.1242 | -0.67 |
| 354.1144 | [M+H]^+^ | C_20_H_17_FNO_4_ | B | 354.1136 | 2.17 |
| 341.0786 | [M+H]^+^ | C_19_H_14_FO_5_ | C | 341.0820 | -9.86 |
| 294.0922 | [M+H]^+^ | C_18_H_13_FNO_2_ | D | 294.0925 | -0.81 |
| 208.0767 | [M+H]^+^ | C_11_H_11_FNO_2_ | E | 208.0768 | -0.59 |

**Supplementary Fig. 40** High-resolution MS^2^ spectra of F-protopine, displaying potential fragment formulas. Source data are provided as a Source Data file.


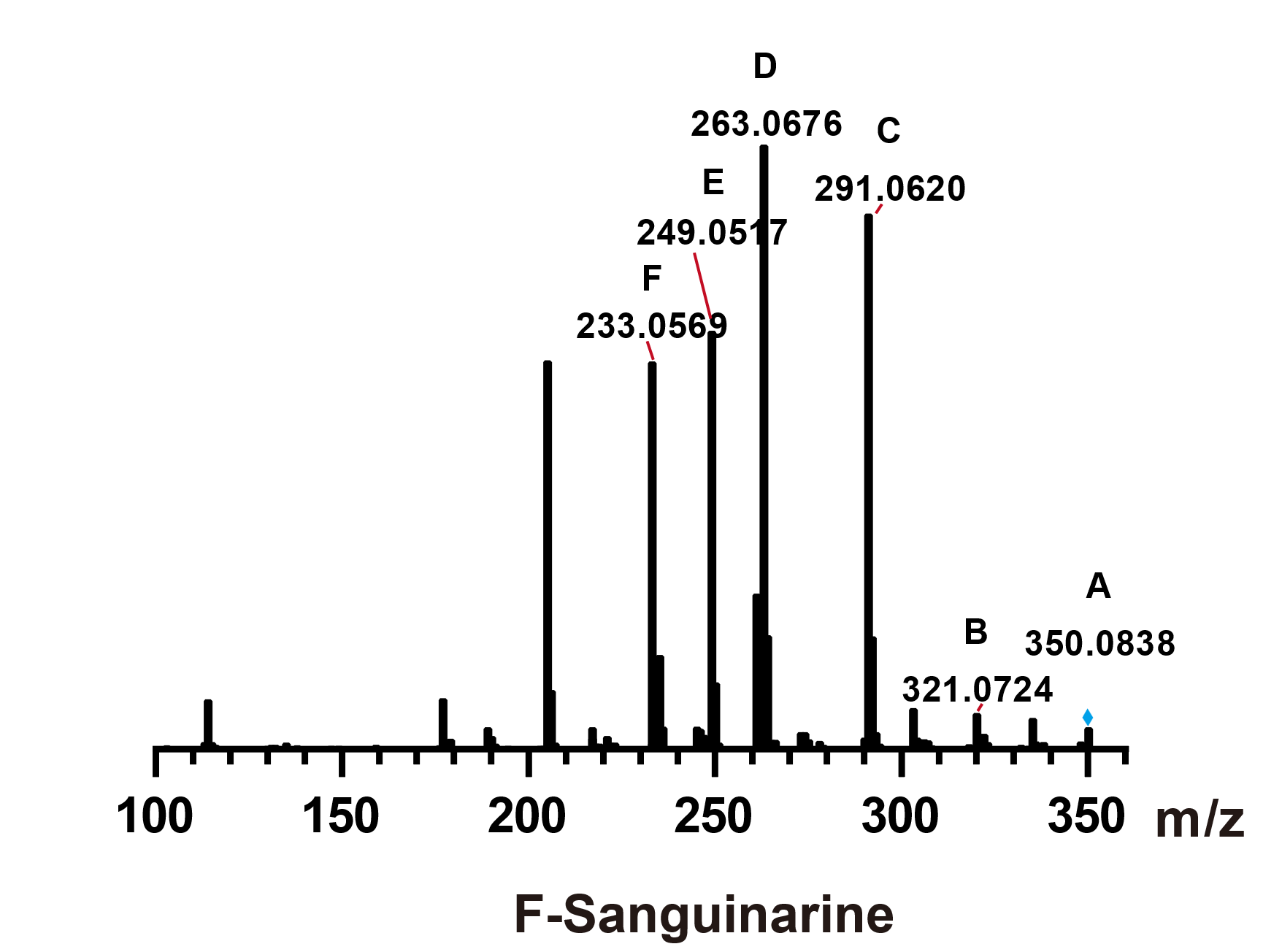


| ***m/z* (observed)** | **Species** | **Predicted formula** | **Ion Assignment** | ***m/z* (theoretical)** | **△*m/z* (ppm)** |
| --- | --- | --- | --- | --- | --- |
| 350.0838 | [M]^+^ | C_20_H_13_FNO_4_ | A | 350.0823 | 0.6 |
| 321.0724 | [M]^+^ | C_19_H_12_FNO_3_ | B | 321.0796 | -22.27 |
| 291.0620 | [M]^+^ | C_18_H_10_FNO_2_ | C | 291.0690 | -23.47 |
| 263.0676 | [M]^+^ | C_17_H_10_FNO | D | 263.0741 | -23.35 |
| 249.0517 | [M]^+^ | C_16_H_8_FNO | E | 249.0584 | -26.92 |
| 233.0569 | [M]^+^ | C_16_H_8_FN | F | 233.0635 | -28.25 |

**Supplementary Fig. 41** High-resolution MS^2^ spectra of F-sanguinarine, displaying potential fragment formulas. Source data are provided as a Source Data file.


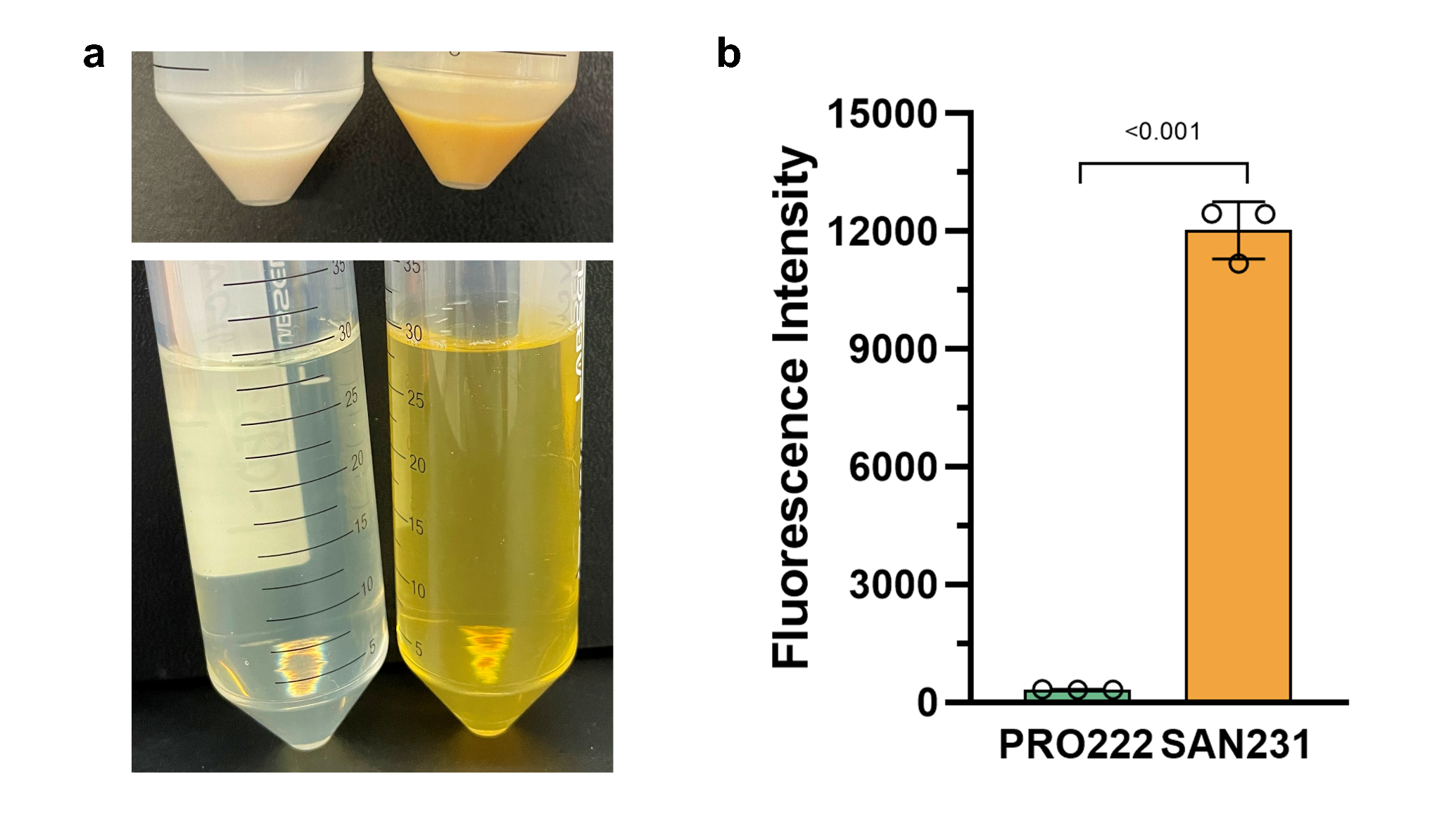
**Supplementary Fig. 42** The conjugate structure of sanguinarine allows visible fluorescence. Comparison of supernatant color (a) and fluorescence intensity (b) between PRO222 and SAN231 strains after 30 mL culture medium fermentation in shake flasks. Data are presented as mean ± s.d. (n = 3 biologically independent samples). Significance was calculated using the unpaired Student’s t test (two-sided). Source data are provided as a Source Data file.

**Supplementary Tables**

**Supplementary Table 1** The pros and cons of GAL4M9 and SIMTeGES-GAL4.

| **Systems** | **GALM9** | **SIMTeGES-GAL4** |
| --- | --- | --- |
| **Mechanisms** | Temperature-sensitive mutant obtained by directed evolution | Inserting temperature-sensitive intein into the host proteins |
| **Advantages** | Lower leakage expression level | Stronger transcriptional activation activity |
|  |  | Suitability for temperature-controlled expression of various target proteins in different host systems |
| **Disadvantages** | Temperature dependence is associated with a trade-off in transcriptional activation capacity, resulting in lower transcriptional activation activity | Slightly higher leakage expression level |
|  | Limited to regulating the expression of target genes under the control of GAL promoters in yeast |  |

**Supplementary Table 2** Summary of the retention time, precursor and collision energy (eV) for the benzylisoquinoline alkaloids derivatives detected in Targeted MS/MS mode LC-qTOF MS.

| **Compound** | **Retention time (min)** | **Delta Ret. time (min)** | **Precursor** | **Collision energy (eV)** | **Window Width** |
| --- | --- | --- | --- | --- | --- |
| F-Norcoclaurine | 1.54 | 0.5 | 290.1185 | 10, 20, 40 | 50 |
| Cl-Norcoclaurine | 1.59 | 0.5 | 306.0886 | 10, 20, 40 | 50 |
| I-Norcoclaurine | 1.62 | 0.5 | 398.0240 | 10, 20, 40 | 50 |
| diF-Norcoclaurine | 1.57 | 0.5 | 308.1090 | 10, 20, 40 | 50 |
| F-Reticuline | 1.64 | 0.5 | 348.1600 | 10, 20, 40 | 50 |
| Cl-Reticuline | 1.69 | 0.5 | 364.1301 | 10, 20, 40 | 50 |
| F-Scoulerine | 1.62 | 0.5 | 346.1445 | 10, 20, 40 | 50 |
| F-Cheilanthifoline | 1.72 | 0.5 | 344.1288 | 10, 20, 40 | 50 |
| F-Ptotopine | 1.76 | 0.5 | 372.1235 | 10, 20, 40 | 50 |
| F-Sanguinarine | 1.80 | 0.5 | 350.0838 | 10, 20, 40 | 50 |

**Supplementary Table 3** Summary of the isotopic distribution of F-sanguinarine detected in Targeted MS/MS mode LC-qTOF MS.

| **m/z**  **(observed)** | **Species** | **Predicted formula** | **Isotope** |
| --- | --- | --- | --- |
| 350.0838 | Precursor A, [M]^+^ | C_20_H_13_FNO_4_ | 351.0816 |
| 321.0724 | Fragment B, [M]^+^ | C_19_H_12_FNO_3_ | 322.0756, 323.0881, 324.0878 |
| 291.0620 | Fragment C, [M]^+^ | C_18_H_10_FNO_2_ | 292.0659, 293.0751, 294.0868 |
| 263.0676 | Fragment D, [M]^+^ | C_17_H_10_FNO | 264.0724, 265.0808 |
| 249.0517 | Fragment E, [M]^+^ | C_16_H_8_FNO | 250.0549, 251.0631 |
| 233.0569 | Fragment F, [M]^+^ | C_16_H_8_FN | 234.0607, 235.0203 |

**Supplementary Table 4** List of sgRNA plasmids for genome integration of heterologous gene expression cassettes and knocking out endogenous genes.

| Plasmid | Description | Spacer sequences (5’-3’) |
| --- | --- | --- |
| pRS423-Δ*GAL4* | 2μ; HIS3; Amp; SNR52p-SpSgΔ*GAL4*-SUP4t | ATCTTGTAAAGGATAGCCAA |
| pRS423-Δ*GAL80* | 2μ; HIS3; Amp; SNR52p-SpSgΔ*GAL80*-SUP4t | GATAGTGATAGCTATCCAAG |
| pRS423-IntS11 | 2μ; HIS3; Amp; SNR52p-SpSgIntS11-SUP4t | GAACAAGAACAACAAACTCC |
| pRS423-IntL4 | 2μ; HIS3; Amp; SNR52p-SpSgIntL4-SUP4t | TAGTGCACTTACCCCACGTT |
| pRS423-IntS10 | 2μ; HIS3; Amp; SNR52p-SpSgIntS10-SUP4t | CATGAGCAGCCACTGTATCG |
| pRS423-IntS4 | 2μ; HIS3; Amp; SNR52p-SpSgIntS4-SUP4t | ACACGTTTGTGGTTATAAGG |
| pRS423-IntS6 | 2μ; HIS3; Amp; SNR52p-SpSgIntS6-SUP4t | ACGTATGGTTGTAAAAAGCA |
| pRS423-IntL5 | 2μ; HIS3; Amp; SNR52p-SpSgIntL5-SUP4t | CACTTGTCAAACAGAATATA |
| pRS423-Δ*HFD1* | 2μ; HIS3; Amp; SNR52p-SpSgΔ*HFD1*-SUP4t | GAAGAATCAGATACTCTCCG |
| pRS423-Δ*PsBBE* | 2μ; HIS3; Amp; SNR52p-SpSgΔ*PsBBE*-SUP4t | ATGTTGTCTGAACAACCAGG |
| pRS423-Δ*CFS-STS* | 2μ; HIS3; Amp; SNR52p-SpSgΔ*CFS-STS*-SUP4t | TCACAAGAAGTCAATCAGAG |
| pRS423-IntG11 | 2μ; HIS3; Amp; SNR52p-SpSgIntG11-SUP4t | AATCTGGAGATAAGGCAACG |
| pRS423-Δ*DBOX-McP6H* | 2μ; HIS3; Amp; SNR52p-SpSgΔ*DBOX-McP6H*-SUP4t | GGTGGTTTTCAATTGCAAGG |
| pRS423-IntL2 | 2μ; HIS3; Amp; SNR52p-SpSgIntL2-SUP4t | GTAATATTGTCTTGTTTCCC |
| pRS423-Δ*ARI1* | 2μ; HIS3; Amp; SNR52p-SpSgΔ*ARI1*-SUP4t | GGATTGCATAGTTGACACCG |
| pRS423-IntL7 | 2μ; HIS3; Amp; SNR52p-SpSgIntL7-SUP4t | AATCCGAACAACAGAGCATA |
| pRS423-IntG17 | 2μ; HIS3; Amp; SNR52p-SpSgIntG17-SUP4t | GAAATTATATAAAACACATG |
| pRS423-IntL9 | 2μ; HIS3; Amp; SNR52p-SpSgIntL9-SUP4t | GCGCCACAGTTTCAAGGGTC |
| pRS423-IntG19 | 2μ; HIS3; Amp; SNR52p-SpSgIntG19-SUP4t | AGATCTTGCGAAATACTGGG |
| pRS423-IntG16 | 2μ; HIS3; Amp; SNR52p-SpSgIntG16-SUP4t | TATATAATGAATACACATGG |
| pRS423-IntG22 | 2μ; HIS3; Amp; SNR52p-SpSgIntG22-SUP4t | TCACACGAATGAGAATTGGG |
| pRS423-IntG21 | 2μ; HIS3; Amp; SNR52p-SpSgIntG21-SUP4t | TCAAGGGGTTGCATATAGGG |
| pRS423-IntG20 | 2μ; HIS3; Amp; SNR52p-SpSgIntG20-SUP4t | TAATCAGTCTAACACCCCGG |
| pRS423-IntG26 | 2μ; HIS3; Amp; SNR52p-SpSgIntG26-SUP4t | GAGAAAATAAAAAAAATATG |
| pRS423-IntG15 | 2μ; HIS3; Amp; SNR52p-SpSgIntG15-SUP4t | GAGGACAGCGTGAATCACAA |

**Supplementary Table 5** Summary of the retention time, fragment voltage (V), transition and collision energy (eV) for the benzylisoquinoline alkaloids detected in MRM mode LC-QQQ MS.

| **Compound** | **Retention time (min)** | **Fragment voltage (V)** | **Transition** | **Collision energy(eV)** | **Qualitative ions** | **Collision energy(eV)** |
| --- | --- | --- | --- | --- | --- | --- |
| *(S)*-norcoclaurine | 1.597 | 120 | 272.1→107.0 | 30 | 161.0 | 20 |
| *(S)*-reticuline | 1.663 | 110 | 330.0→192.0 | 18 | 137.0 | 36 |
| *(S)*-scoulerine | 1.689 | 120 | 328.0→178.0 | 28 | 162.9 | 54 |
| *(S)*-cheilanthifoline | 1.724 | 140 | 326.0→178.0 | 32 | 162.9 | 52 |
| *(S)*-stylopine | 1.793 | 120 | 324.0→176.0 | 35 | 149.0 | 26 |
| protopine | 1.772 | 140 | 354.0→148.9 | 28 | 247.0 | 40 |
| sanguinarine | 1.817 | 135 | 332.0→274.1 | 39 | 317.0 | 36 |

**Supplementary Table 6** Summary of the retention time, fragment voltage (V), precursor and collision energy (eV) for the benzylisoquinoline alkaloids derivatives detected in PRO mode LC-QQQ MS.

| **Compound** | **Retention time (min)** | **Fragment voltage (V)** | **Precursor** | **Collision energy(eV)** | **Characteristic fragments** |
| --- | --- | --- | --- | --- | --- |
| F-Norcoclaurine | 1.602 | 130 | 290 | 21 | 273, 179, 161, 125, 107 |
| Cl-Norcoclaurine | 1.624 | 120 | 306.5 | 23 | 289.5, 160.5, 141.5 |
| I-Norcoclaurine | 1.649 | 120 | 398 | 25 | 381, 233, 161 |
| diF-Norcoclaurine | 1.608 | 120 | 308 | 20 | 291, 179, 125 |
| F-Reticuline | 1.669 | 120 | 348 | 22 | 317, 192, 155 |
| Cl-Reticuline | 1.734 | 135 | 364.5 | 27 | 335.5, 192, 171.5 |
| F-Scoulerine | 1.739 | 130 | 346 | 26 | 178 |
| F-Cheilanthifoline | 1.755 | 140 | 344 | 28 | 178, 163 |
| F-Stylopine | 1.715 | 140 | 342 | 30 | 194, 176, 149 |
| F*-N-*Methylscoulerine | 1.757 | 140 | 360 | 28 | 194, 178 |
| F*-N-*Methylcheilanthifoline | 1.747 | 130 | 358 | 27 | 192, 190, 177 |
| F*-N-*Methylstylopine | 1.825 | 145 | 356 | 27 | 190, 188 |
| Protopine | 1.772 | 140 | 354 | 30 | 275, 247, 149 |
| F-Ptotopine | 1.806 | 140 | 372 | 30 | 293, 167 |
| Sanguinarine | 1.839 | 135 | 332 | 41 | 317, 274, 246 |
| F-Sanguinarine | 1.740 | 150 | 350 | 43 | 264, 292 |
