# Supplementary material for: Intein-mediated temperature control for complete biosynthesis of sanguinarine and its halogenated derivatives in yeast
Source: Nat Commun. 2024 Jun 19;15:5238. doi: 10.1038/s41467-024-49554-w (PMC11186835; doi:10.1038/s41467-024-49554-w)
Supplement: Supplementary file 3 — Description of Additional Supplementary Files [file 41467_2024_49554_MOESM3_ESM.pdf]

Description of Additional Supplementary Files

Supplementary Data 1 Yeast strains and cells used in this study.

Supplementary Data 2. List of plasmids used in this study.

Supplementary Data 3. List of heterologous genes used in this study.

Supplementary Data 4. List of gene coding sequences used in this study.

Supplementary Data 5. List of oligonucleotides used in this study.
