# Supplementary material for: Intein-mediated temperature control for complete biosynthesis of sanguinarine and its halogenated derivatives in yeast
Source: Nat Commun. 2024 Jun 19;15:5238. doi: 10.1038/s41467-024-49554-w (PMC11186835; doi:10.1038/s41467-024-49554-w)
Supplement: Supplementary file 9 — Reporting Summary [file 41467_2024_49554_MOESM9_ESM.pdf]

nature portfolio

Corresponding author(s): Jiazhang Lian

Last updated by author(s): Jun 4, 2024

Reporting Summary

Nature Portfolio wishes to improve the reproducibility of the work that we publish. This form provides structure for consistency and transparency in reporting. For further information on Nature Portfolio policies, see our Editorial Policies and the Editorial Policy Checklist.

Statistics

For all statistical analyses, confirm that the following items are present in the figure legend, table legend, main text, or Methods section.

n/a	Confirmed	
☐	☒	The exact sample size (*n*) for each experimental group/condition, given as a discrete number and unit of measurement
☐	☒	A statement on whether measurements were taken from distinct samples or whether the same sample was measured repeatedly
☐	☒	The statistical test(s) used AND whether they are one- or two-sided<br>*Only common tests should be described solely by name; describe more complex techniques in the Methods section.*
☒	☐	A description of all covariates tested
☒	☐	A description of any assumptions or corrections, such as tests of normality and adjustment for multiple comparisons
☐	☒	A full description of the statistical parameters including central tendency (e.g. means) or other basic estimates (e.g. regression coefficient) AND variation (e.g. standard deviation) or associated estimates of uncertainty (e.g. confidence intervals)
☐	☒	For null hypothesis testing, the test statistic (e.g. *F*, *t*, *r*) with confidence intervals, effect sizes, degrees of freedom and *P* value noted<br>*Give P values as exact values whenever suitable.*
☒	☐	For Bayesian analysis, information on the choice of priors and Markov chain Monte Carlo settings
☒	☐	For hierarchical and complex designs, identification of the appropriate level for tests and full reporting of outcomes
☒	☐	Estimates of effect sizes (e.g. Cohen's *d*, Pearson's *r*), indicating how they were calculated

Our web collection on statistics for biologists contains articles on many of the points above.

Software and code

Policy information about availability of computer code

Data collection	1. Cell imaging: confocal images were captured using a Nikon Ti microscope with a 100× PlanAPO lens (NA 1.49), equipped with four diode lasers (405, 488, 555, and 639nm).<br>2.Folw cytometery: Attune NxT flow cytometer (Thermo Fisher Scientific)<br>3.Immune blot signaling detection: ChemiDoc MP Imaging System/100-240VAC/50-60HZ/250VF8A/5*20mm<br>4. Mass spectrum: the 6470 triple quadrupole LC/MS system in production mode and the 6545 high-resolution mass spectrometry LC/Q-TOF in Targeted MS/MS mode<br>5.Fluorescence Intensity: Tecan Infinite 200 PRO microplate reader (Trading AG, Switzerland).
Data analysis	1. GraphPad Prism 8 software was used to analyze statistical significance for all comparison studies using Student's t-test (unpaired and two-tailed), one-way ANOVA, or two-way ANOVA. Multiple comparisons were conducted.<br>2. Image Lab 5.2.1 (BioRad) was used to analyse the western bolt signal.<br>3. The LC-QQQ and LC-MS-TOF data were analyzed using MassHunter software (Agilent Technologies) (version 10.0).<br>4. Benchling CRISPR online tool (https://benchling.com/crispr) was used for gRNA design.

For manuscripts utilizing custom algorithms or software that are central to the research but not yet described in published literature, software must be made available to editors and reviewers. We strongly encourage code deposition in a community repository (e.g. GitHub). See the Nature Portfolio guidelines for submitting code & software for further information.

Data

Policy information about availability of data

All manuscripts must include a data availability statement. This statement should provide the following information, where applicable:

- Accession codes, unique identifiers, or web links for publicly available datasets
- A description of any restrictions on data availability
- For clinical datasets or third party data, please ensure that the statement adheres to our policy

All the data supporting the findings in this study is available within the Main Text and Supplementary Information. Source data is provided as a Source Data file. All the plasmids containing splicing intein-mediated temperature-responsive gene expression system (SIMTeGES), the refactored sanguinarine pathway, and the lycopene pathway, that support the findings of this study are available from the corresponding author Jiazhang Lian (jzlian@zju.edu.cn) upon reasonable requests.

Research involving human participants, their data, or biological material

Policy information about studies with human participants or human data. See also policy information about sex, gender (identity/presentation), and sexual orientation and race, ethnicity and racism.

Reporting on sex and gender	No information on sex and gender because there was no human research conducted.
Reporting on race, ethnicity, or other socially relevant groupings	No information on race, ethnicity, or other socially relevant groupings because there was no human research conducted.
Population characteristics	No information on population characteristics because there was no human research conducted.
Recruitment	No recruitment method was used because there was no human research conducted.
Ethics oversight	No ethics oversight was implemented because there was no human research conducted.

Note that full information on the approval of the study protocol must also be provided in the manuscript.

Field-specific reporting

Please select the one below that is the best fit for your research. If you are not sure, read the appropriate sections before making your selection.

☒ Life sciences ☐ Behavioural & social sciences ☐ Ecological, evolutionary & environmental sciences

For a reference copy of the document with all sections, see nature.com/documents/nr-reporting-summary-flat.pdf

Life sciences study design

All studies must disclose on these points even when the disclosure is negative.

Sample size	Sample sizes, comprising a minimum of three biological replicates, were carefully chosen to align with our vast expertise and established protocols in yeast engineering. The selected sample sizes (N=3, N=4, or N=5) were not only based on our extensive experience but also maintained consistency with the throughput requirements of various experimental techniques. These techniques encompassed strain cultivation, measurement of sugar and metabolite concentrations, as well as quantification of cell densities.
Data exclusions	No data was excluded from the manuscript.
Replication	All experiments were conducted in biological triplicates, quadruplicates, or quintuplicates. A biological replicate in the context of our experimentation is one yeast colony streaked from the strain stock (not one individual culture from the same inoculum, as this represents a technical replicate). We confirmed that all attempts at replication were successful in this study.
Randomization	Randomization was not performed as the nature of the genetic and biochemical experiments and the associated data does not potentiate human bias influencing the final conclusions. In our particular study, we have focused on investigating the relationship between a specific independent variable (e.g., time, genotype, and initial OD) and the dependent variable (e.g., concentration and cell growth OD), without considering the influence of other variables. By simplifying the study design in this way, we aimed to gain a clearer understanding of the direct impact of the independent variable on the outcome, thus allowing for a more focused analysis and interpretation of the results.
Blinding	Blinding was not performed as the nature of the genetic and biochemical experiments and the associated data generated does not potentiate human bias influencing the final conclusions.

Reporting for specific materials, systems and methods

We require information from authors about some types of materials, experimental systems and methods used in many studies. Here, indicate whether each material, system or method listed is relevant to your study. If you are not sure if a list item applies to your research, read the appropriate section before selecting a response.

Materials & experimental systems

n/a	Involved in the study
☐	☒ Antibodies
☐	☒ Eukaryotic cell lines
☒	☐ Palaeontology and archaeology
☒	☐ Animals and other organisms
☒	☐ Clinical data
☒	☐ Dual use research of concern
☒	☐ Plants

Methods

n/a	Involved in the study
☒	☐ ChIP-seq
☐	☒ Flow cytometry
☒	☐ MRI-based neuroimaging

Antibodies

Antibodies used: DYKDDDDK Tag Mouse Monoclonal antibody (1:10000, 66008-4-Ig, proteintech), HRP-labeled Goat Anti-Mouse IgG (H+L) (1:2000, A0216, Beyotime)

Validation: All the antibodies are commercially-available. The antibodies have been validated by the manufacturers, which can be found in the corresponding manufacturers' websites. No additional validation experiments were carried out.

Eukaryotic cell lines

Policy information about cell lines and Sex and Gender in Research

Cell line source(s): S. cerevisiae BY4741 (commercially available)
P. pastoris GS115 (commercially available)
HEK 293T (commercially available)

Authentication: Cell lines were used without further authentication.

Mycoplasma contamination: Cells lines were not tested for mycoplasma contamination.

Commonly misidentified lines (See ICLAC register): No commonly misidentified lines are included.

Plants

Seed stocks: *Report on the source of all seed stocks or other plant material used. If applicable, state the seed stock centre and catalogue number. If plant specimens were collected from the field, describe the collection location, date and sampling procedures.*

Novel plant genotypes: *Describe the methods by which all novel plant genotypes were produced. This includes those generated by transgenic approaches, gene editing, chemical/radiation-based mutagenesis and hybridization. For transgenic lines, describe the transformation method, the number of independent lines analyzed and the generation upon which experiments were performed. For gene-edited lines, describe the editor used, the endogenous sequence targeted for editing, the targeting guide RNA sequence (if applicable) and how the editor was applied.*

Authentication: *Describe any authentication procedures for each seed stock used or novel genotype generated. Describe any experiments used to assess the effect of a mutation and, where applicable, how potential secondary effects (e.g. second site T-DNA insertions, mosiacism, off-target gene editing) were examined.*

Flow Cytometry

Plots

Confirm that:

☒ The axis labels state the marker and fluorochrome used (e.g. CD4-FITC).

☒ The axis scales are clearly visible. Include numbers along axes only for bottom left plot of group (a 'group' is an analysis of identical markers).

☒ All plots are contour plots with outliers or pseudocolor plots.

☒ A numerical value for number of cells or percentage (with statistics) is provided.

Methodology

Sample preparation: Pre-cultured strains were transferred to 24 deep-well plates containing 2 mL of YPG medium with an initial OD600=0.2. After

Sample preparation	36h of cultivation at 30 or 25 °C, with agitation at 800r.p.m., cells were harvested by centrifugation at 6,000 r.p.m. for 2 minutes and washed twice with PBS. The monoparametric detection of mCherry fluorescence in each sample was analyzed using a YL2 (561/620nm) filter. A total of 10,0000 cells were counted per sample to determine the percentage of fluorescent cells. GAL4-dINT cells were used to establish the negative boundary. In the subsequent experiments, the same boundary and gating strategy were applied to calculated fluorescent-positive cells.
Instrument	Attune NxT flow cytometer (Thermo Fisher Scientific)
Software	FlowJo 10.8.1.
Cell population abundance	Flow sorting of cells was not involved in this study
Gating strategy	Different groups in which cells with or without mCherry fluorescence signals were used in the gating stratgy.

☒ Tick this box to confirm that a figure exemplifying the gating strategy is provided in the Supplementary Information.
